# Design, Synthesis and Biological Evaluation of Novel 5*H*-Chromenopyridines as Potential Anti-Cancer Agents

**DOI:** 10.3390/molecules200917152

**Published:** 2015-09-17

**Authors:** Souvik Banerjee, Jin Wang, Susan Pfeffer, Dejian Ma, Lawrence M. Pfeffer, Shivaputra A. Patil, Wei Li, Duane D. Miller

**Affiliations:** 1 Department of Pharmaceutical Sciences, College of Pharmacy, University of Tennessee Health Science Center, 847 Monroe Avenue, Memphis, TN 38163, USA; E-Mails: sbanerj5@uthsc.edu (S.B.); jwang58@uthsc.edu (J.W.); dma6@uthsc.edu (D.M.); spatil3@uthsc.edu (S.A.P.); wli@uthsc.edu (W.L.); 2 Department of Pathology and Laboratory Medicine, College of Medicine and the Center for Cancer Research, University of Tennessee Health Science Center, 19 S Manassas, Memphis, TN 38163, USA; E-Mails: spfeffer@uthsc.edu (S.P.); lpfeffer@uthsc.edu (L.M.P.)

**Keywords:** glioma, melanoma, chromene, chromenopyridine, anti-proliferative activity

## Abstract

A novel series of 5*H*-chromenopyridines was identified as anticancer agents in our continuing effort to discover and develop new small molecule anti-proliferative agents. Based on our initial lead **SP-6-27** compound, we designed and synthesized novel tricyclic 5*H*-thiochromenopyridine and 5*H*-chromenopyridine analogs to evaluate the impact of an additional ring, as well as conformational flexibility on cytotoxic activity against human melanoma and glioma cell lines. All of the 5*H*-thiochromenopyridines have been achieved in good yields (89%–93%) using a single-step, three-component cyclization without the need for purification. The 5*H*-chromenopyridine analog of the potent 5*H*-thiochromenopyride was obtained in a good yield upon purification. All newly-prepared 5*H*-thiochromenopyridines showed good to moderate cytotoxicity against three melanoma and two glioma cell lines (3–15 μM). However, the 5*H*-chromenopyridine analogue that we prepared in our laboratory lost cytotoxic activity. The moderate cytotoxic activity of 5*H*-thiochromenopyridines shows the promise of developing chromenopyridines as potential anticancer agents.

## 1. Introduction

On a global scale, cancer is a major public health problem [1]. At present, one quarter of all deaths in the USA are caused by cancer [2]. A wide variety of anti-cancer agents has been described for the treatment of various kinds of cancers, including glioma and melanoma. However, many of these agents show a major failure in terms of drug resistance and toxicity. Hence, there is an emerging need for the discovery and development of novel anticancer agents to overcome drug resistance and toxicity issues.

**Figure 1:**
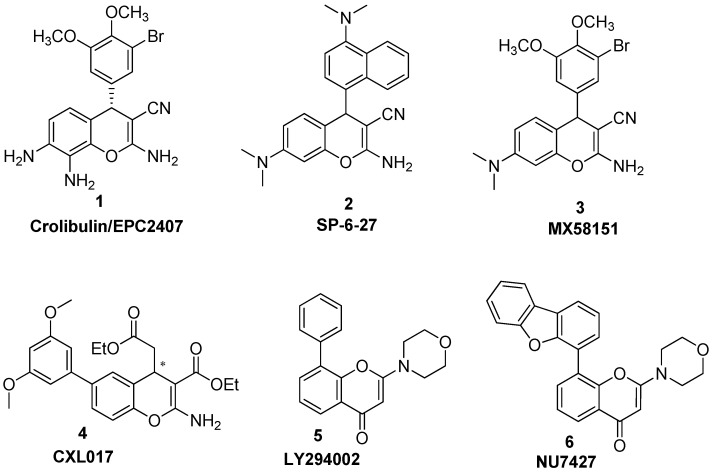
Chromene-based anti-cancer agents.

Recently, chromene analogs have emerged as potent anticancer agents, and crolibulin is currently in a phase II clinical trial for anaplastic thyroid cancer with the National Cancer Institute (NCI) [3,4] (Figure 1).

**Figure 2:**
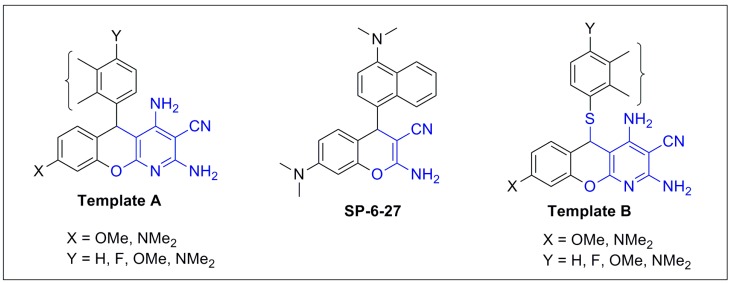
Hypothesized chromenopyridines.

Our recent anticancer screening program identified **SP-6-27** as one of the potent anticancer agents *in vitro* [5]. The 60 National Cancer Institute Developmental Therapeutic preclinical cancer cell lines screen indicated that **SP-6-27** has consistent anti-proliferative activity against nine major cancer cell lines and has been selected for further *in vivo* testing [6]. Although there are a number of articles demonstrating bicyclic chromenes as anti-cancer agents [4,5,7,8,9,10,11,12,13], there are only a few reports on tricyclic chromenopyridines as anti-proliferative candidates [14,15]. Thus, the aim of the present study is to determine the potential of the novel tricyclic chromenopyridines as anticancer agents. In light of our recent success with **SP-6-27**, it would be interesting to envisage the biological activities of two different templates of tricyclic chromenopyridines, rigid 5*H*-substituted-chrominopyridines (Template **A**; Figure 2) and relatively flexible 5*H*-sustituted-thiochromenopyridines (Template **B**; Figure 2).

We thought that additional flexibility in Template **B** type molecules, conferred by the inserted sulfur atom, could play a crucial role in biological activity.

Briefly, starting with **SP-6-27**, we were interested in determining the effect of an additional ring (Template **A**-type molecules; Figure 2) and a combination of both an additional ring, as well as flexibility (Template **B**-type candidates; Figure 2) on cytotoxic activity on human melanoma and glioma cell lines. Here, we report the optimal structural requirements of the linear tricyclic chromenopyridines for biological activity.

## 2. Results and Discussion

**Scheme 1:**
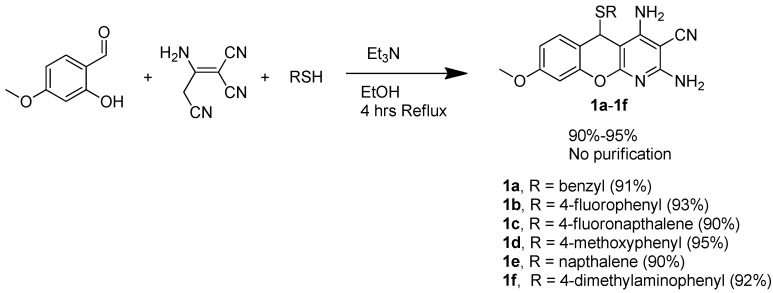
Classical synthesis of 5*H*-substituted-thiochromenopyridines.

The development of a single-step, multicomponent reaction strategy to prepare a small library of compounds was of considerable interest. Anderson *et al*. first reported the synthesis of 5*H*-substituted-chromenopyridines (Template **A**-type; Figure 2) utilizing two-step condensation of 3*H*-substituted-phenol with an arylmethylidene derivative of malonitrile and malonitrile, respectively [16]. In the following year, Melekhin *et al*. reported a single-step, two-component preparation of 5*H*-substituted-chromenopyridines (Template **A**-type; Figure 2) via condensation of resorcinol with an arylmethylidene derivative of the malonitrile dimer [17]. To the best of our knowledge, Evdokimov *et al*. are the first to demonstrate a single-step, three-component cyclization technique, which utilized salicylaldehyde, thiophenol and malonitrile, to prepare 5*H*-substituted-thiochromenopyridines (Template **B**-type; Figure 2) [18]. Recently, Ghomi *et al*. demonstrated tin oxide nanoparticles, as well as zirconium nanoparticle as efficient catalysts to promote four-component cyclization of salicylaldehyde, thiols and two equivalent malonitriles, resulting in 5*H*-substituted-thiochromenopyridines in a good yield [19,20]. Here, we report an efficient, as well as fast three-component cyclization reaction, which works both under normal reflux and microwave irradiation, to prepare 5*H*-substituted-thiochromenopyridines, as shown in Scheme 1. The synthetic procedure for the preparation of six 5*H*-substituted-thiochromenopyridines (**1a**–**1f**), consisting of the C^8^-methoxy substituent, is presented in Scheme 1.

**Scheme 2:**
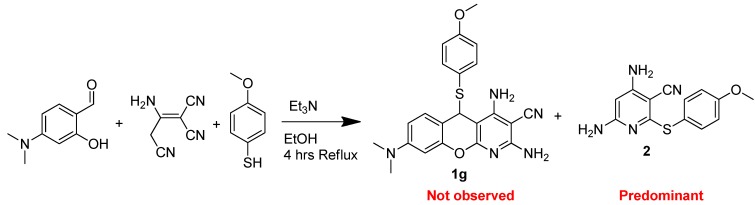
Synthesis of 5*H*-substituted-thiochromenopyridine.

Briefly, 4-methoxy-salicylaldehyde was subjected to a three-component cyclization reaction with 2-amino-1,1,3-propenetricarbonitrile (malonitrile dimer) and the aromatic thiol of interest, employing a catalytic amount of trimethylamine in ethanol under a refluxing condition. The desired thiochromenopyridines were obtained in great yields upon simple recrystallization, ruling out the need for a tedious purification process. At this point, we made an effort to prepare a few thiochromenopyridines consisting of C^8^-dimethylamino substituents utilizing 4-dimethylamino-salisaldehyde following the same synthetic method. Scheme 2 demonstrates our effort to prepare C^8^-dimethylamino-thiochromenopyridines.

**Scheme 3:**
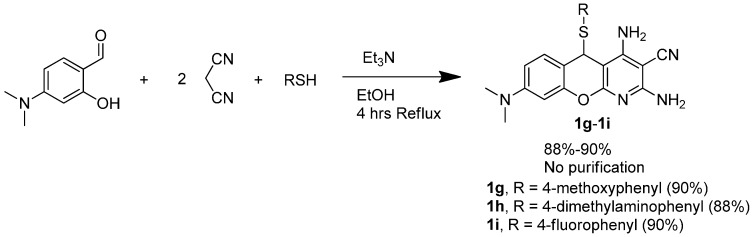
Classical synthesis of 5*H*-substituted-thiochromenopyridines.

To our surprise, 4-dimethylamino-salisaldehyde was observed as unreacted and 4-methoxy-thiophenol reacted with the malonitrile dimer to afford the undesired, highly-substituted pyridine derivative **2**. At this point, we thought of employing two equivalents of malonitriles, as demonstrated by Evdokimov *et al.* [18], instead of the malonitrile dimer. Interestingly, the reactions worked as desired. Scheme 3 demonstrates our second and successful attempt to prepare three thiochromenopyridines (**1g**–**1i**) consisting of the C^8^-dimethylamino substituent.

**Scheme 4:**
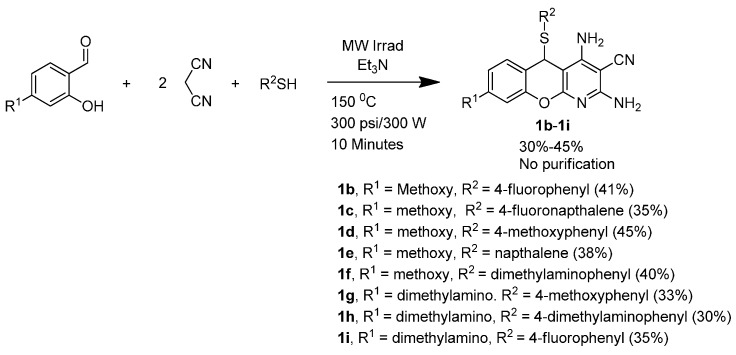
Microwave-assisted synthesis of 5*H*-substituted-thiochromenopyridines.

This finding suggests that unlike the malonitrile dimer, two equivalents of malonitrile work both for electron-deficient and electron-rich salisaldehyde. In continuation, we made an effort to conduct the three-component cyclization under microwave irradiation utilizing two equivalents of malonitrile in order to further shorten the reaction time. Our effort to achieve eight 5*H*-substituted-thiochromenopyridines is summarized in Scheme 4. Under controlled microwave irradiation, the reaction time is further shortened to 10 min. However, the yield is compromised to 30%–45%.

**Figure 3:**
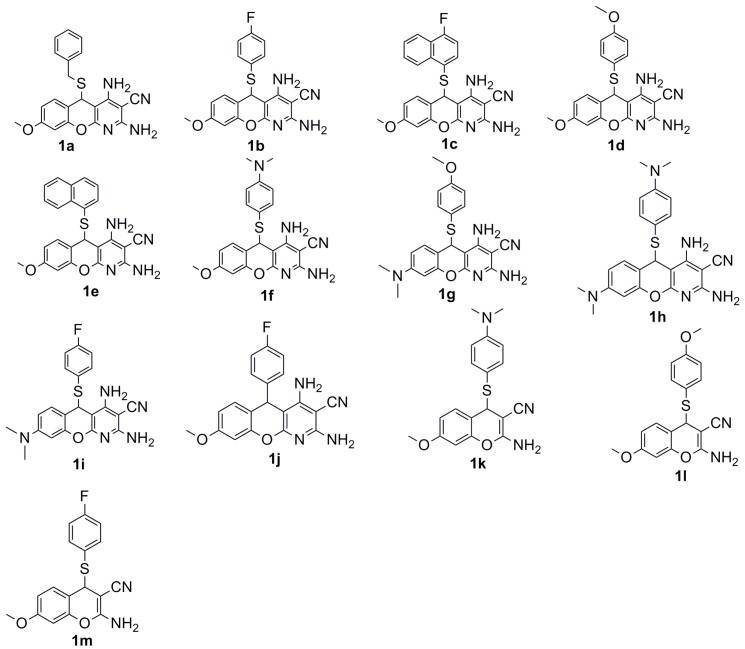
Synthesized chromenopyridines and chromenes.

In this study, ten 5*H*-substituted-chromenopyridines (**1a**–**1j**), as well as three 4*H*-substituted chromenes (**1k**–**1m**) were designed and synthesized, as shown in Figure 3.

**Scheme 5:**
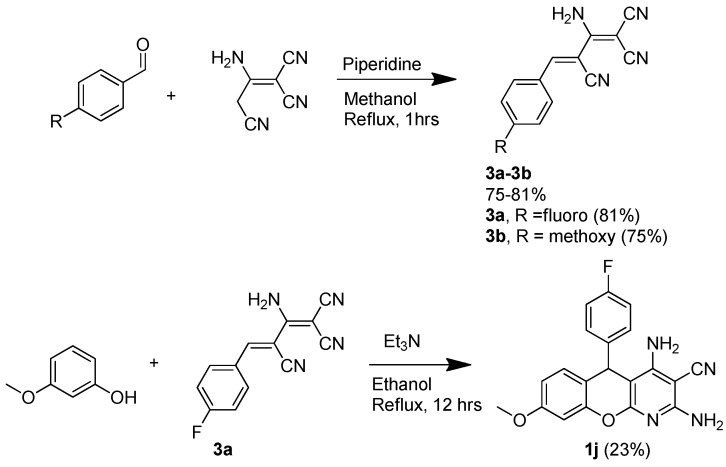
Synthesis of 5H-substituted-chromenopyridine.

Upon obtaining nine 5*H*-substituted-thiochromenopyridines (**1a**–**1i**), we tested their antiproliferative activity against three melanoma cell lines and two glioma cell lines *in vitro*. Table 1 represent the IC_50_ values of the prepared compounds against three melanoma and two glioma cell lines. Interestingly, all nine compounds are found moderately active against melanoma cell lines, and eight of them are observed as moderately active against glioma cell lines, as indicated by Table 1. Interestingly, Compound **1a**, consisting of a benzyl head group, is tolerated in melanoma cell lines, but not in glioma cell lines. It is noteworthy that the C^8^-methoxy substituent offers better activity in both cell lines compared to the C^8^-dimethylamino substituent. Table 1 suggests that **1b** and **1c** are the two most potent compounds (IC_50_ ~ 3.6 μM) against melanoma. Likewise, **1e** and **1f** are the two most potent for glioma cell lines with IC_50_ values in the 3 μM range. This finding also suggests that the fluoro head group (**1b**, **1c**) is preferentially active against the melanoma cell lines, and the dimethylamino head group (**1f**) is preferentially active against the glioma cell lines. At this point, we decided to prepare Template **A**-type chromenopyridines (Figure 2) that do not consist of flexible carbon-sulfur-carbon bonds. We thought that this may provide insight into the role of flexibility offered by the carbon-sulfur-carbon bonds (**1a**–**1i**) for the biological activity. We chose to prepare Template **A**-type derivatives of **1b** and **1f** based on the observed activity. Scheme 5 demonstrates our effort to prepare rigid 5*H*-substituted-chromenopyridine following the procedure demonstrated by Melekhin *et al*. [17] However, only **1j**, a rigid analogue of **1b**, worked out in our hands, as summarized in Scheme 5. To our surprise, **1j** was observed as inactive against all melanoma and glioma cell lines. Hence, this finding suggests that the flexibility offered by carbon-sulfur-carbon bonds (**1a**–**i1**) is critical for cytotoxic activity. However, we have learned from **SP-6-27** that rigid chromenes are active. To the best of our knowledge, there is no report on the cytotoxicity of the 4*H*-substituted-thiochromenes.

**Table 1:** Anti-proliferative activity of prepared chromenopyridine and chromene analogues.

Compound ID	IC_50_ ± SEM (µM)				
	A375	WM164	MDA-MB-435	SJG2	MT330
**1a**	6.4 ± 0.8	7.5 ± 1.2	7.4 ± 0.8	> 30	> 30
**1b**	6.7 ± 1.5	3.5 ± 1.2	3.7 ± 0.8	4.7 ± 5	6.1 ± 1.9
**1c**	6.3 ± 0.7	3.6 ± 0.6	4.1 ± 0.4	5.6 ± 0.8	5.6 ± 0.8
**1d**	7.0 ± 0.9	6.6 ± 1.0	6.5 ± 0.6	5.2 ± 1.4	4.6 ± 0.1
**1e**	5.3 ± 0.7	5.7 ± 1.4	6.0 ± 0.8	3.1 ± 2.8	4.5 ± 0.9
**1f**	5.7 ± 0.4	5.6 ± 0.6	7.1 ± 0.5	3.3 ± 2.7	5.2 ± 0.0
**1g**	7.2 ± 1.4	9.7 ± 2.7	9.7 ± 2.6	5.1 ± 0.1	5.4 ± 0.0
**1h**	16.8 ± 0.4	25.9 ± 3.5	19.1 ± 1.3	5.3 ± 0.2	8.3 ± 0.0
**1i**	10.6 ± 0.6	15.0 ± 2.2	8.9 ± 0.7	5.1 ± 0.5	5.1 ± 0.0
**1j**	>30	>30	21.9 ± 4.4	ND	ND
**1k**	>30	>30	>30	ND	ND
**1l**	>30	>30	>30	ND	ND
**1m**	>30	>30	>30	ND	ND
**SP-6-27**	0.08 ± 0.01	0.16 ± 0.03	ND	0.07 ± 0.02	0.05 ± 0.03
**Colchicine**	0.02 ± 0.01	0.03 ± 0.02	ND	NT	NT

Note. ND, activity not detected. The upper limit of activation is 30 µM. NT, not tested against those cell lines.

Hence, it would be interesting to see if the flexibility offered by the bicyclic 4*H*-substituted thiochromenes is tolerated.

**Scheme 6:**
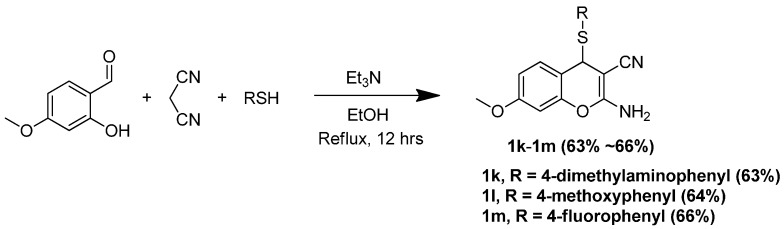
Synthesis of 4*H*-substituted-thiochromenes.

Scheme 6 describes our efforts to prepare three 4*H*-substituted thiochromenes (**1k**–**1m**) following the technique demonstrated by Evdokimov *et al.* [18].

Interestingly, none of the thiochromenes (**1k**–**1m**) were found to be active against both cell lines.

Briefly, our initial findings suggest that the tricyclic thiochromenopyridines (**1a**–**1i**) have potential to be developed as novel anti-cancer agents. However, the activity is limited to the lower micromolar range. Hence, additional research is needed to optimize the activity of these compounds.

## 3. Experimental Section

### 3.1. Chemistry

#### 3.1.1. General

All reagents were purchased from Sigma-Aldrich Chemical Co. (St. Louis, MO, USA) and were used without further purification. The solvents for moisture-sensitive reactions were freshly distilled, and the reactions were carried out in an argon atmosphere. Routine thin-layer chromatography (TLC) was performed on aluminum-backed Uniplates (Analtech, Newark, DE, USA). Nuclear magnetic resonance spectra were obtained on a Varian Inova-500 spectrometer (Agilent, Santa Clara, CA, USA) or a Bruker Avance III 400 MHz (Bruker BioSpin, Billerica, MA, USA) spectrometer. Chemical shifts are reported as parts per million (ppm) relative to TMS in CDCl_3_. Mass spectra were collected on a Bruker ESQUIRE electrospray/ion trap instrument (Bruker Daltonics Inc., Billerica, MA, USA) in positive and negative ion modes. Elemental analysis (C, H, N) was performed by Atlantic Microlab, Inc. (Norcross, GA, USA), and results were within ±0.4% of the theoretical values for the formula given. Yields refer to purified products.

#### 3.1.2. General Procedure for the Synthesis of 5*H*-Substituted-Thiochromenopyridines (**1a**–**1f**) under Regular Reflux

An amount of 2-amino-1,1,3-propenetricarbonitrile (1 mmol), desired thiophenol or thionapthanol (1 mmol) and triethyl amine (0.1 mmol) were added in a solution of 4-methoxy-salisaldehyde (1 mmol) in 7 mL EtOH. The resulting mixture was allowed to reflux over 4 h, at which point the product precipitates out of the solution. The resulting precipitate was filtered and dried under vacuum. The residue was then dissolved in 3 mL DMF. The insoluble particles were filtered off. A volume of 4 mL H_2_O was then poured into the resulting filtrate, leading to the precipitation of the pure product out of the solution. The precipitates were filtered off and dried under vacuum, leading to the pure 5*H*-substituted-thiochromenopyridines (89% to 91%) as light yellowish solids.

*2,4-Diamino-5-(benzylthio)-8-methoxy-5H-chromeno[2,3-b]pyridine-3-carbonitrile* (**1a**): Compound **1a** was prepared following the general procedure for the preparation of Compounds **1a**–**1f**. An amount of 355 mg (0.91 mmol, 91%) **1a** was obtained as a light yellowish solid. ^1^H-NMR (DMSO-*d*_6_, 400 MHz): δ 7.26 (m, 1H), 7.17 (m, 3H), 7.05 (m, 2H), 6.79 (m, 3H), 6.67 (m, 1H), 6.53 (bs, 2H), 5.44 (s,1H), 3.78 (s, 3H), 3.45 (m, 2H). ^13^C-NMR (DMSO-*d*_6_, 100 MHz): δ 159.7, 159.6, 159.3, 159.5, 151.8, 137.5, 129.3, 128.6, 128.1, 126.5, 116.4, 114.4, 111.1, 101.0, 87.1, 70.6, 55.4, 38.1, 32.7. MP = 193.4 °C. Anal. calcd. for C_21_H_18_N_4_O_2_S C, 64.60; H, 4.65; N, 14.35. Found C, 64.42; H, 4.72; N, 14.40.

*2,4-Diamino-5-((4-fluorophenyl)thio)-8-methoxy-5H-chromeno[2,3-b]pyridine-3-carbonitrile* (**1b**): Compound **1b** was prepared following the general procedure for the preparation of Compounds **1a**–**1f**. An amount of 367 mg (0.93 mmol, 93%) **1b** was obtained as a light yellowish solid. ^1^H-NMR (DMSO-*d*_6_, 400 MHz): δ 7.12 (m, 1H), 6.97 (m, 4H), 6.75 (m, 3H), 6.50 (bs, 2H), 6.40 (m, 1H), 5.68 (s, 1H), 3.73 (s, 3H). ^13^C-NMR (DMSO-*d*_6_, 100 MHz): δ 164.0, 161.5, 159.6 , 159.3, 156.3, 1518, 138.4, 129.5, 126.5, 116.5, 115.3, 113.5, 110.9, 100.3, 86.3, 70.3, 55.4, 43.0. ^19^F-NMR (DMSO-*d*_6_, 400 MHz) δ −111.72 (external standard, trifluoroacetic acid, δ −75.56). MP = 219.4 °C. Anal. calcd. for C_20_H_15_FN_4_O_2_S C, 60.90; H, 3.83; N, 14.20. Found C, 61.01; H, 4.02; N, 14.26.

*2,4-Diamino-5-((4-fluoronaphthalen-1-yl)thio)-8-methoxy-5H-chromeno[2,3-b]pyridine-3-carbonitrile* (**1c**): Compound **1c** was prepared following the general procedure for the preparation of Compounds **1a**–**1f**. An amount of 400 mg (0.90 mmol, 90%) **1c** was obtained as a light yellowish solid. ^1^H-NMR (DMSO-*d*_6_, 400 MHz): δ 7.94 (d, 1H, *J* = 7.76 Hz), 7.80 (m, 1H), 7.50 (t, 1H, *J* = 7.76 Hz), 7.29 (t, 1H, *J* = 7.76 Hz), 7.20 (d, 1H, *J* = 7.76 Hz), 7.09 (m, 1H), 7.0 (bs,2H). 6.85 (m, 1H), 6.71 (m, 1H), 6.31 (bs, 2H), 6.12 (m, 1H), 5.82 (s, 1H), 3.66 (s, 3H). ^13^C-NMR (DMSO-*d*_6_, 100 MHz): δ 160.2, 159.5, 159.4, 157.6, 156.4, 152.0, 137.2, 136.7, 129.4, 126.6, 126.3, 125.8, 124.1, 123.0, 119.7, 116.6, 113.4, 111.1, 109.1, 100.1, 86.4, 70.4, 55.4, 44.0. ^19^F-NMR (DMSO-*d*_6_, 400 MHz) δ −119.58 (external standard, trifluoroacetic acid, δ −75.56). MP = 201.9 °C. Anal. calcd. for C_24_H_17_FN_4_O_2_S C, 64.85; H, 3.86; N, 12.60. Found C 64.65; H, 3.86; N, 12.76.

*2,4-Diamino-8-methoxy-5-((4-methoxyphenyl)thio)-5H-chromeno[2,3-b]pyridine-3-carbonitrile* (**1d**): Compound **1d** was prepared following the general procedure for the preparation of Compounds **1a**–**1f**. An amount of 388 mg (0.95 mmol, 95%) **1d** was obtained as a light yellowish solid. ^1^H-NMR (DMSO-*d*_6_, 400 MHz): δ 7.09 (d, 1H, *J* = 8.14 Hz), 6.88 (bs, 2H), 6.69 (m, 5H), 6.47 (bs, 2H), 6.39 (m, 1H), 5.59 (s, 1H), 3.74 (s, 3H), 3.70 (s, 3H). ^13^C-NMR (DMSO-*d*_6_, 100 MHz): δ 160.0, 159.6, 159.5, 159.1, 156.3, 151.8, 137.7, 129.5, 121.3, 116.6, 113.9, 113.8, 110.7, 100.4, 86.6, 70.3, 55.4, 55.1, 42.6. MP = 200.7 °C. Anal. calcd. for C_21_H_18_N_4_O_3_S C, 62.05; H, 4.46; N, 13.78. Found C, 62.21; H, 4.61; N, 13.88.

*2,4-Diamino-8-methoxy-5-(naphthalen-1-ylthio)-5H-chromeno[2,3-b]pyridine-3-carbonitrile* (**1e**): Compound **1e** was prepared following the general procedure for the preparation of Compounds **1a**–**1f**. An amount of 385 mg (0.90 mmol, 90%) **1e** was obtained as a light yellowish solid. ^1^H-NMR (DMSO-*d*_6_, 400 MHz): δ 7.82 (m, 3H), 7.39 (m, 1H), 7.19 (m, 3H), 6.96 (m, 3H), 6.67 (m, 1H), 6.3 (bs, 2H), 6.12 (m, 1H), 5.83 (s, 1H), 3.66 (s, 3H). ^13^C-NMR (DMSO-*d*_6_, 100 MHz): δ 160.0, 159.6, 159.5, 159.1, 156.3, 151.8, 137.7, 129.5, 121.3, 116.6, 113.9, 113.8, 110.7, 100.4, 86.6, 70.3, 55.4, 55.1, 42.6. MP = 199.1 °C. Anal. calcd. for C_24_H_18_N_4_O_2_S C, 67.59; H, N, 13.14. Found C, 67.52; H, 4.34; N, 13.13.

*2,4-Diamino-5-((4-(dimethylamino)phenyl)thio)-8-methoxy-5H-chromeno[2,3-b]pyridine-3-carbonitrile* (**1f**): Compound **1f** was prepared following the general procedure for the preparation of Compounds **1a**–**1f**. An amount of 386 mg (0.92 mmol, 92%) **1f** was obtained as a light yellowish solid. ^1^H-NMR (DMSO-*d*_6_, 400 MHz): δ 7.06 (d, 1H, *J* = 8.60 Hz), 6.81 (bs, 2H), 6.70 (m, 1H), 6.55 (m, 2H), 6.41 (m, 5H), 5.46 (s, 1H), 3.73 (s, 3H), 2.84 (s, 6H). ^13^C-NMR (DMSO-*d*_6_, 100 MHz): δ 159.6, 159.4, 160.0, 156.3, 151.7, 150.5, 137.2, 129.5, 116.6, 115.1, 114.2, 111.6, 110.5, 100.4, 87.0, 70.3, 55.3, 42.4. MP = 187.0 °C. Anal. calcd. for C_22_H_21_N_5_O_2_S C, 62.99; H, 5.05; N, 16.69. Found C, 62.86; H, 5.15; N, 16.72.

#### 3.1.3. General Procedure for the Synthesis of 5*H*-Substituted-Thiochromenopyridines (**1g**–**1i**)

An amount of malonitrile (2 mmol), desired thiophenol (1 mmol) and triethyl amine (0.1 mmol) were added in a solution of 4-dimethylamino-salisaldehyde (1 mmol) in 7 mL EtOH. The resulting mixture was allowed to reflux over 4 h, at which point the product precipitates out of the solution. The resulting precipitate was filtered off and dried under vacuum. The residue was then dissolved in 3 mL DMF. The insoluble particles were filtered off. A volume of 4 mL H_2_O was then poured into the resulting filtrate, leading to the precipitation of the pure product out of the solution. The precipitates were filtered off and dried under vacuum, leading to the pure 5*H*-substituted-thiochromenopyridines (**1g**–**1i**, 89%–91%) as light pinkish solids.

*2,4-Diamino-8-(dimethylamino)-5-((4-methoxyphenyl)thio)-5H-chromeno[2,3-b]pyridine-3-carbonitrile* (**1g**): Compound **1g** was prepared following the general procedure for the preparation of Compounds **1g**–**1i**. An amount of 388 mg (0.90 mmol, 92%) **1g** was obtained as a light pinkish solid. ^1^H-NMR (DMSO-*d*_6_, 400 MHz): δ 6.97 (d, 1H, *J* = 8.87 Hz), 6.79 (bs, 2H), 6.68 (m, 4H), 6.51 (m, 1H), 6.40 (m, 2H), 6.07 (m, 1H), 5.53 (s, 1H), 3.69 (s, 3H), 2.87 (s, 6H). ^13^C-NMR (DMSO-*d*_6_, 100 MHz): δ 160.0, 159.8, 159.4, 156.2, 151.8, 150.4, 137.6, 129.0, 121.8, 116.7, 113.7, 109.1, 108.5, 98.2, 87.1, 70.1, 55.1, 43.0, 40.0. MP = 172.9 °C. Anal. calcd. for C_22_H_21_N_5_O_2_S C, 62.99; H, 5.05; N, 16.69. Found C, 63.01; H, 5.08; N, 16.64.

*2,4-Diamino-8-(dimethylamino)-5-((4-(dimethylamino)phenyl)thio)-5H-chromeno[2,3-b]pyridine-3-carbonitrile* (**1h**): Compound **1h** was prepared following the general procedure for the preparation of Compounds **1g**–**1i**. An amount of 388 mg (0.88 mmol, 88%) **1h** was obtained as a light pinkish solid. ^1^H-NMR (DMSO-*d*_6_, 400 MHz): δ 6.95 (d, 1H, *J* = 8.49 Hz), 6.73 (bs, 2H), 6.58 (m, 2H), 6.50 (m, 1H), 6.39 (m, 4H), 6.10 (m, 1H), 5.42 (s, 1H), 2.86 (m, 12H). ^13^C-NMR (DMSO-*d*_6_, 100 MHz): δ 162.3, 160.0, 159.3, 156.2, 151.7, 150.3, 150.2, 137.2, 129.1, 116.7, 115.7, 111.6, 109.5, 108.5, 98.3, 87.4, 70.1, 42.8. MP = 140.5 °C. Anal. calcd. for C_23_H_24_N_6_OS C, 63.87; H, 5.59; N, 19.43. Found C, 63.89; H, 5.62; N, 19.39.

*2,4-Diamino-8-(dimethylamino)-5-((4-fluorophenyl)thio)-5H-chromeno[2,3-b]pyridine-3-carbonitrile* (**1i**): Compound **1i** was prepared following the general procedure for the preparation of Compounds **1g**–**1i**. An amount of 368 mg (0.90 mmol, 90%) **1i** was obtained as a light pinkish solid. ^1^H-NMR (DMSO-*d*_6_, 400 MHz): δ 6.97 (m, 3H), 6.85 (bs, 2H), 6.78 (m, 2H), 6.52 (m, 1H), 6.43 (bs, 2H), 6.08 (s, 1H), 5.63 (s, 1H), 2.87 (s, 6H). ^13^C-NMR (DMSO-*d*_6_, 100 MHz): δ 163.9, 161.4, 160.0, 159.5, 156.2, 152.0, 150.5, 138.3, 128.9, 126.9, 116.6, 115.3, 115.0, 108.8, 98.1, 86.8, 70.2, 43.5. ^19^F-NMR (DMSO-*d*_6_, 400 MHz) δ −112.06 (external standard, trifluoroacetic acid, δ −75.56). MP = 189.3 °C. Anal. calcd. for C_21_H_18_FN_5_OS C, 61.90; H, 4.45; N, 17.19. Found C, 61.95; H, 4.54; N, 17.26.

#### 3.1.4. General Procedure for the Synthesis of 5*H*-Substituted-Thiochromenopyridines (**1a**–**1i**) under Microwave Irradiation

A mixture of 4-substituted-salisaldehyde (1 equivalent), malonitrile (2 equivalent) and the desired substituted-phenylthiol or naphthol (1 equivalent) was mixed in 3 mL ethanol in a microwave tube. A catalytic amount of trimethylamine (2–4 drops) was added to the solution. The reaction mixture was irradiated at 150 °C using 300 psi pressure and 300 Watts of power over 10 min in CEM Discover. The reaction mixture was cooled down to room temperature; the precipitates were filtered and dried under vacuum. The resulting residue was dissolved in 3 mL DMF. The insoluble particles were removed by filteration. A volume of 4 mL H_2_O was added to the DMF layer, leading to the precipitation of the desired product. The precipitates were filtered and dried under vacuum, resulting in the pure product as a yellowish or a pinkish solid in a decent yield (30%–45%), as reported in Scheme 4. The characterizations of the obtained products (**1a**–**1i**), under microwave irradiation, comply with the ones obtained under regular reflux condition.

*2,4-Diamino-5-(4-fluorophenyl)-8-methoxy-5H-chromeno[2,3-b]pyridine-3-carbonitrile* (**1j**): An amount of 124 mg 3-methoxy-phenol (1 mmol) was added in a solution of 240 mg (*Z*)-2-amino-4-(4-fluorophenyl)buta-1,3-diene-1,1,3-tricarbonitrile (**3a**), prepared following the literature reported procedure, in 15 mL ethanol. The volume of a catalytic amount of piperidine was added to the reaction mixture. The reaction mixture was brought to reflux overnight. The reaction mixture was cooled down, evaporated out, and the crude was purified utilizing 2% MeOH in methylene chloride, resulting in 30 mg (8.3%) product as a yellowish solid. ^1^H-NMR (DMSO-*d*_6_, 400 MHz): δ 7.3 (m, 3H), 7.1 (m, 3H), 6.65 (m, 1H), 6.46 (m, 4H), 5.28 (s, 1H), 3.74 (s, 3H). MP = 184.8 °C. MS (ESI): *m*/*z* calculated [M + H]^+^ 363.37, observed 363.5.

#### 3.1.5. General Procedure for the Preparation of 4*H*-Substituted-Thiochromenes (**1k**–**1m**)

An equimolar mixture of 4-methoxy-salisaldehyde, malonitrile and the desired substituted phenylthiol were mixed in 7 mL EtOH. The reaction mixture was brought to reflux overnight under argon atmosphere. The reaction was allowed to cool down to room temperature, resulting in the precipitation of the product. The product was filtered off and recrystallized in diethyl ether, leading to the pure 4*H*-substituted-thiochromenes (**1k**–**1m**) in good yields.

*2-Amino-4-((4-(dimethylamino)phenyl)thio)-7-methoxy-4H-chromene-3-carbonitrile (**1k**)*: Compound **1k** was prepared following the general procedure for the preparation of Compounds **1k**–**1m**. An amount of 223 mg (0.63 mmol, 63%) **1k** was obtained as a light yellowish solid. ^1^H-NMR (DMSO-*d*_6_, 400 MHz): δ 7.18 (m, 1H), 6.9 (bs, 2H), 6.75 (m, 3H), 6.5 (m, 2H), 6.29 (m, 1H), 4.95 (s, 1H), 3.72 (s,3H), 2.75 (s, 6H). ^13^C-NMR (DMSO-*d*_6_, 100 MHz): δ 161.7, 159.0, 150.5, 149.7, 137.3, 129.8, 120.0, 115.0, 113.6, 111.7, 100.0, 55.4, 54.2, 46.5. MP = 136.4 °C. Anal. calcd. for C_19_H_19_N_3_O_2_S C, 64.57; H, 5.42; N, 11.89. Found C, 63.82; H, 5.56; N, 11.27.

*2-Amino-7-methoxy-4-((4-methoxyphenyl)thio)-4H-chromene-3-carbonitrile* (**1l**): Compound **1l** was prepared following the general procedure for the preparation of Compounds **1k**–**1m**. An amount of 217 mg (0.637 mmol, 64%) **1l** was obtained as a light yellowish solid. ^1^H-NMR (DMSO-*d*_6_, 400 MHz): δ 7.33 (m, 1H), 6.92 (m, 4H), 6.76 (m, 3H), 6.32 (m, 2H), 5.12 (s, 1H), 3.72 (m,6H). ^13^C-NMR (DMSO-*d*_6_, 100 MHz): δ 161.8, 160.0, 159.1, 149.8, 137.7, 129.8, 121.2, 119.9, 114.0, 113.3, 111.4, 100.0, 55.4, 55.1, 54.0, 46.7. MP = 136.0 °C. Anal. calcd. for C_18_H_16_N_2_O_3_S C, 63.51; H, 4.74; N, 8.23. Found C, 63.34; H, 4.92; N, 8.33.

*2-Amino-4-((4-fluorophenyl)thio)-7-methoxy-4H-chromene-3-carbonitrile* (**1m**): Compound **1m** was prepared following the general procedure for the preparation of Compounds **1k**–**1m**. An amount of 217 mg (0.637 mmol, 64%) **1m** was obtained as a light yellowish solid. ^1^H-NMR (DMSO-*d*_6_, 400 MHz): δ 7.28 (m, 1H), 7.1 (m, 2H), 7.0 (m, 4H), 6.8 (m, 1H), 6.3 (m, 1H), 5.3 (s, 1H), 3.75 (s, 3H). ^13^C-NMR (DMSO-*d*_6_, 100 MHz): δ 164.1, 162.0, 159.3, 149.9, 138.4, 129.8, 126.4, 119.6, 115.6, 112.9, 111.6, 100.0, 55.4, 53.8, 47.0. ^19^F-NMR (DMSO-*d*_6_, 400 MHz) δ −111.73 (external standard, trifluoroacetic acid, δ −75.56). MP = 124.6 °C. Anal. calcd. for C_17_H_13_FN_2_O_2_S C, 62.18; H, 3.99; N, 8.53. Found C, 62.31; H, 4.17; N, 8.66.

### 3.2. Biology

#### Cell Culture and Cytotoxicity Assay

##### Melanoma

Human A375, WM164 and MDA-MB-435 melanoma cells were cultured in 10% fetal bovine serum supplemented with DMEM medium with 1% antibiotics at 37 °C 5% carbon oxide. The cell viability was determined by the MTS assay as described previously [21,22]. In brief, 5000 cells were seeded into each well of 96-well plates overnight. Then, a serial dilution of the compounds dissolved in culture medium was used to treat the cells for 48 h. After that, the medium was aspirated, and MTS agent was added to detect the cell density in each well by reading the optical absorbance at 490 nm.

##### Glioma

Human MT330 and SJG2 glioma cells were grown in DMEM with 10% fetal bovine serum. Cells were maintained in the presence of penicillin (100 IU/mL) and streptomycin (100 mg/mL) at 37 °C with 5% CO_2_. Cell viability was determined using the MTT assay. Cells (1000–2000/well) were plated overnight in 96-well plates and treated with various concentrations of the drugs for 4 days. MTT reagent (10 μL/well) was added to the cells; cells were solubilized, and the optical density at 570 nm was measured.

## 4. Conclusions

We have identified a series of novel thiochromenopyridines as potential cytotoxic agents against a number of human cancer cell lines. To the best of our knowledge, this is the first report of thiochromenopyridines as potential anticancer agents. This study reveals that the addition of a third ring to **SP-6-27**, to give **1j**, leads to no detectable activity (ND). In continuation, insertion of a sulfur atom, as in **1k**, **1l**, **1m**, leads to no detectable activity. Finally, insertion of a sulfur atom, as well as a third ring, as in **1a**–**1i**, provided activity, although in the 3–15 µM range. Briefly, this study suggests that the flexible carbon-sulfur-carbon bonds in 5*H*-substituted-thiochromenopyridines are important for cytotoxicity. We propose that the observed diminished activity of 5*H*-substituted-chromenopyridine (**1j**) is due to the absence of flexible-carbon-sulfur-carbon bonds. However, the same flexibility is responsible for the observed inactivity of 4*H*-substituted-thiochromens (**1k–1m**). To summarize, flexible carbon-sulfur-carbon bonds are required for the activity of tricyclic chromenopyridines, but not for the bicyclic chromenes. In addition, compounds with C^8^-methoxy substituents (**1b**, **1c**, **1e** and **1f**) offer better activity as compared to the C^8^-dimethylamino substituents (**1h**, **1i** and **1j**). The good to moderate activity observed with the thio-tricyclic chromenopyridines established them as initial hits, and further research will be needed to develop chromenopyridines as potential leads.

## Supplementary Materials

Supplementary materials can be accessed at: http://www.mdpi.com/1420-3049/20/09/17153/s1.

## References

[1] Jemal A., Bray F., Center M.M., Ferlay J., Ward E., Forman D. (2011). Global cancer statistics. CA Cancer J. Clin..

[2] Siegel R., Ma J., Zou Z., Jemal A. (2014). Cancer statistics, 2014. CA Cancer J. Clin..

[3] Lu Y., Chen J., Xiao M., Li W., Miller D.D. (2012). An Overview of Tubulin Inhibitors That Interact with the Colchicine Binding Site. Pharm. Res..

[4] Patil S.A., Patil R., Pfeffer L.M., Miller D.D. (2013). Chromenes: Potential new chemotherapeutic agents for cancer. Future Med. Chem..

[5] Patil S.A., Wang J., Li X.S., Chen J., Jones T.S., Hosni-Ahmed A., Patil R., Seibel W.L., Li W., Miller D.D. (2012). New substituted 4*H*-chromenes as anticancer agents. Bioorg. Med. Chem. Lett..

[6] Patil S.A., Pfeffer L.M., Miller D.D. (2014). Gliomas: Classification, Symptoms, Treatment and Prognosis.

[7] Kemnitzer W., Drewe J., Jiang S., Zhang H., Wang Y., Zhao J., Jia S., Herich J., Labreque D., Storer R. (2004). Discovery of 4-aryl-4*H*-chromenes as a new series of apoptosis inducers using a cell- and caspase-based high-throughput screening assay. 1. Structure-activity relationships of the 4-aryl group. J. Med. Chem..

[8] Kemnitzer W., Kasibhatla S., Jiang S., Zhang H., Zhao J., Jia S., Xu L., Crogan-Grundy C., Denis R., Barriault N. (2005). Discovery of 4-aryl-4*H*-chromenes as a new series of apoptosis inducers using a cell- and caspase-based high-throughput screening assay. 2. Structure-activity relationships of the 7- and 5-, 6-, 8-positions. Bioorg. Med. Chem. Lett..

[9] Shestopalov A.M., Litvinov Y.M., Rodinovskaya L.A., Malyshev O.R., Semenova M.N., Semenov V.V. (2012). Polyalkoxy Substituted 4*H*-Chromenes: Synthesis by Domino Reaction and Anticancer Activity. ACS Comb. Sci..

[10] Gourdeau H., Leblond L., Hamelin B., Desputeau C., Dong K., Kianicka I., Custeau D., Boudreau C., Geerts L., Cai S.X. (2004). Antivascular and antitumor evaluation of 2-amino-4-(3-bromo-4,5-dimethoxy-phenyl)-3-cyano-4*H*-chromenes, a novel series of anticancer agents. Mol. Cancer Ther..

[11] Azizmohammadi M., Khoobi M., Ramazani A., Emami S., Zarrin A., Firuzi O., Miri R., Shafiee A. (2013). 2*H*-chromene derivatives bearing thiazolidine-2,4-dione, rhodanine or hydantoin moieties as potential anticancer agents. Eur. J. Med. Chem..

[12] Mansouri K., Khodarahmi R., Foroumadi A., Mostafaie A., Mohammadi Motlagh H. (2011). Anti-angiogenic/proliferative behavior of a “4-aryl-4*H*-chromene” on blood vessel’s endothelial cells: A possible evidence on dual “anti-tumor” activity. Med. Chem. Res..

[13] Hussain M.K., Ansari M.I., Yadav N., Gupta P.K., Gupta A.K., Saxena R., Fatima I., Manohar M., Kushwaha P., Khedgikar V. (2014). Design and synthesis of ER_α_/ER_β_ selective coumarin and chromene derivatives as potential anti-breast cancer and anti-osteoporotic agents. RSC Adv..

[14] Thapa U., Thapa P., Karki R., Yun M., Choi J.H., Jahng Y., Lee E., Jeon K.H., Na Y., Ha E.M. (2011). Synthesis of 2,4-diaryl chromenopyridines and evaluation of their topoisomerase I and II inhibitory activity, cytotoxicity, and structure—activity relationship. Eur. J. Med. Chem..

[15] Al-Said M., Ghorab M., Nissan Y. (2012). Dapson in heterocyclic chemistry, part VIII: Synthesis, molecular docking and anticancer activity of some novel sulfonylbiscompounds carrying biologically active 1,3-dihydropyridine, chromene and chromenopyridine moieties. Chem. Cent. J..

[16] Anderson D.R., Hegde S., Reinhard E., Gomez L., Vernier W.F., Lee L., Liu S., Sambandam S., Snider P.A., Masih L. (2005). Aminocyanopyridine inhibitors of mitogen activated protein kinase-activated protein kinase 2 (MK-2). Bioorg. Med. Chem. Lett..

[17] Melekhin E.A., Bardasov I.N., Ershov O.V., Eremkin A.V., Kayukov Y.S., Nasakin O.E. (2006). Synthesis of 5-Aryl-2,4-diamino-8-hydroxy-5*H*-chromeno[2,3-*b*]pyridine-3-carbonitriles. Russ. J. Org. Chem..

[18] Evdokimov N.M., Kireev A.S., Yakovenko A.A., Antipin M.Y., Magedov I.V., Kornienko A. (2007). One-Step Synthesis of Heterocyclic Privileged Medicinal Scaffolds by a Multicomponent Reaction of Malononitrile with Aldehydes and Thiols. J. Org. Chem..

[19] Safaei-Ghomi J., Kiani M., Ziarati A., Shahbazi-Alavi H. (2014). Highly efficient synthesis of benzopyranopyridines via ZrP_2_O_7_ nanoparticles catalyzed multicomponent reactions of salicylaldehydes with malononitrile and thiols. J. Sulfur Chem..

[20] Safaei-Ghomi J., Shahbazi-Alavi H., Heidari-Baghbahadorani E. (2014). SnO nanoparticles as an efficient catalyst for the one-pot synthesis of chromeno[2,3-*b*]pyridines and 2-amino-3,5-dicyano-6-sulfanyl pyridines. RSC Adv..

[21] Chen J., Ahn S., Wang J., Lu Y., Dalton J.T., Miller D.D., Li W. (2012). Discovery of novel 2-aryl-4-benzoyl-imidazole (ABI-III) analogues targeting tubulin polymerization as antiproliferative agents. J. Med. Chem..

[22] Wang Z., Chen J., Wang J., Ahn S., Li C.M., Lu Y., Loveless V.S., Dalton J.T., Miller D.D., Li W. (2012). Novel tubulin polymerization inhibitors overcome multidrug resistance and reduce melanoma lung metastasis. Pharm. Res..

