# Supplementary material for: Design, Synthesis and Biological Evaluation of Novel 5H-Chromenopyridines as Potential Anti-Cancer Agents
Source: Molecules. 2015 Sep 17;20(9):17152–65. doi: 10.3390/molecules200917152 (PMC6332407; doi:10.3390/molecules200917152)
Supplement: Supplementary file 1 [file molecules-20-17152-s001.pdf]

Supplementary Materials

^{1}H- ,^{13}C-, and ^{19}F-NMR Spectra

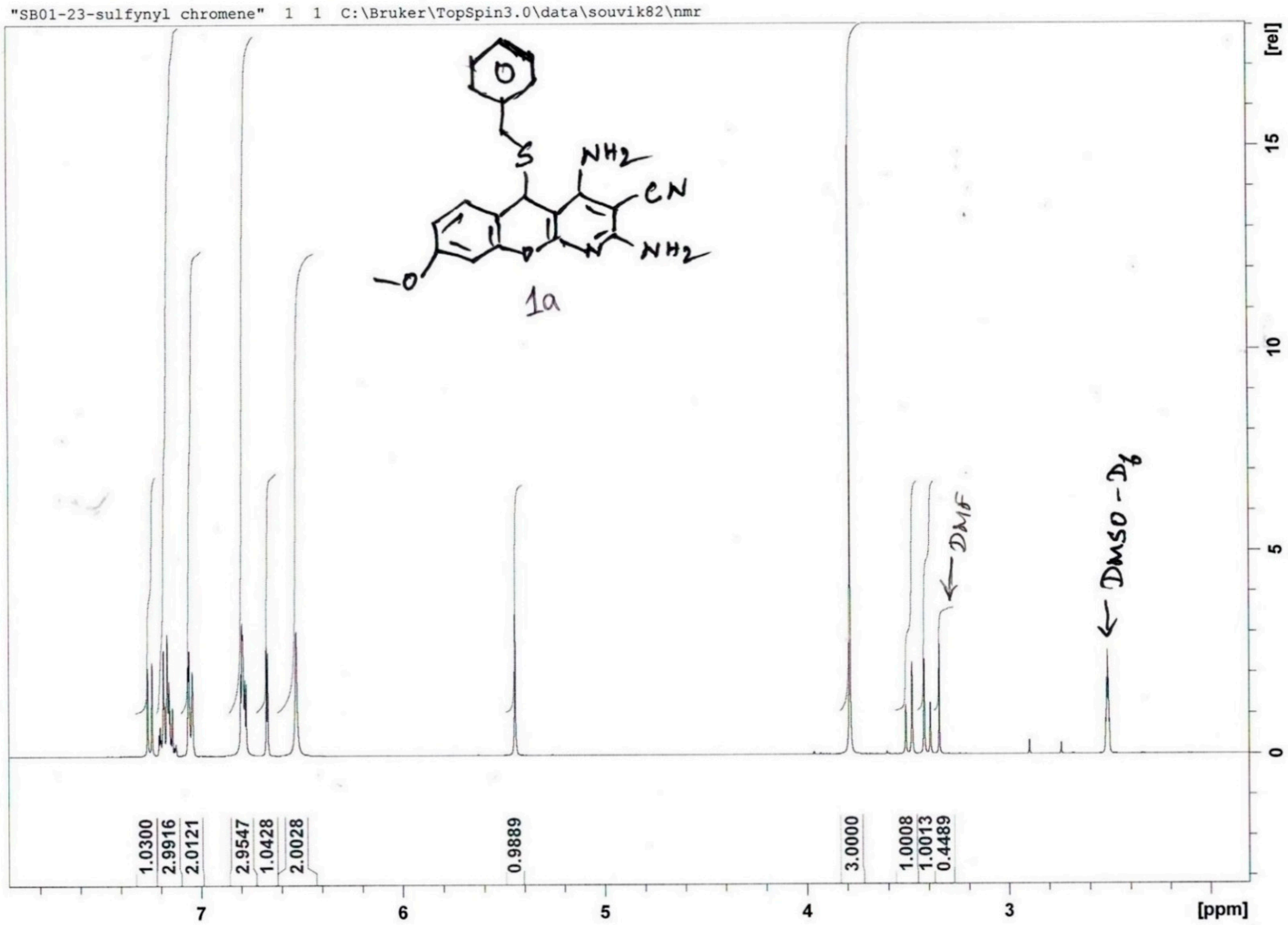

Figure S1. ^{1}H-NMR of **1a**.

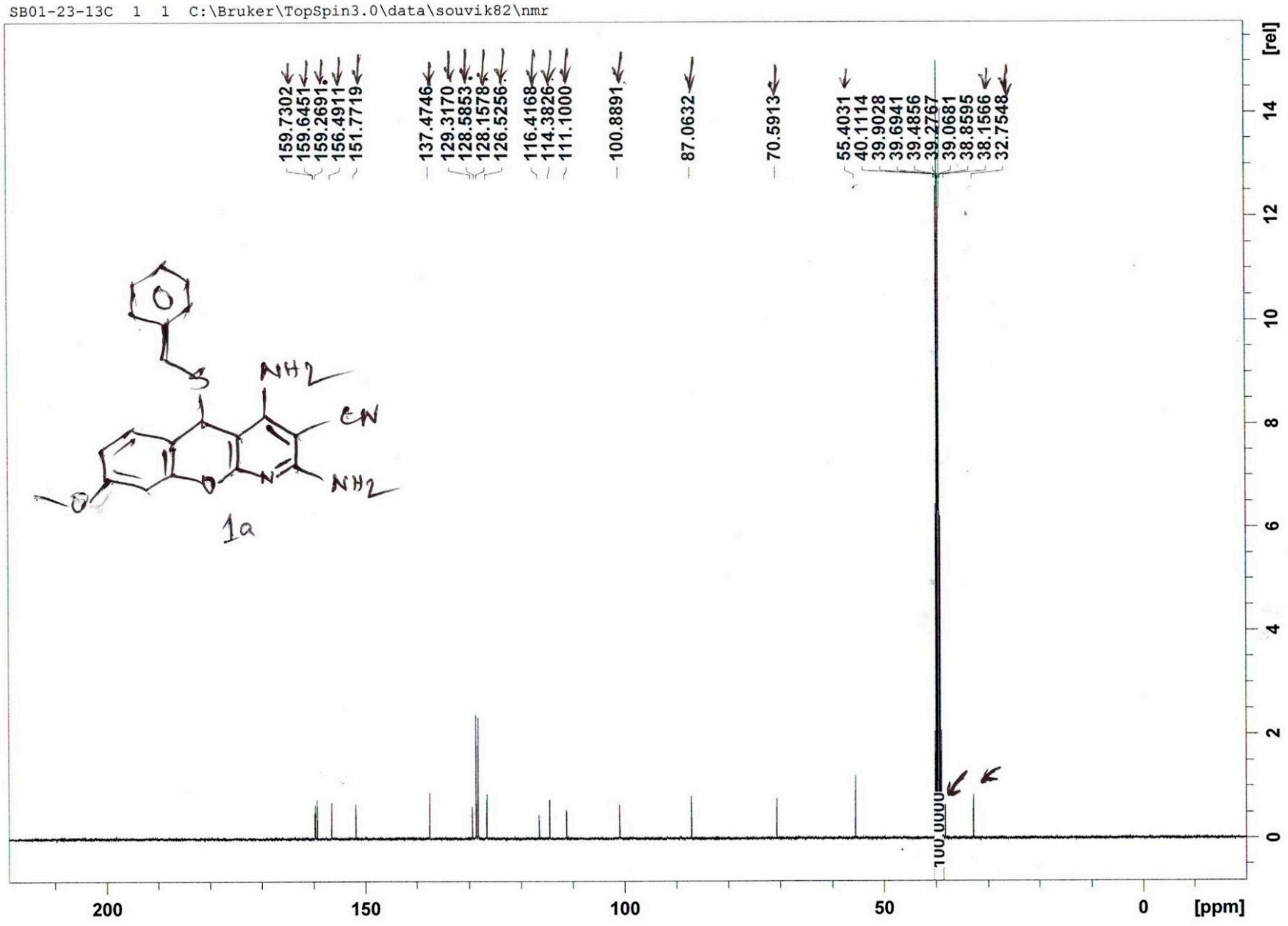

Figure S2. ^{13}C-NMR of **1a**.

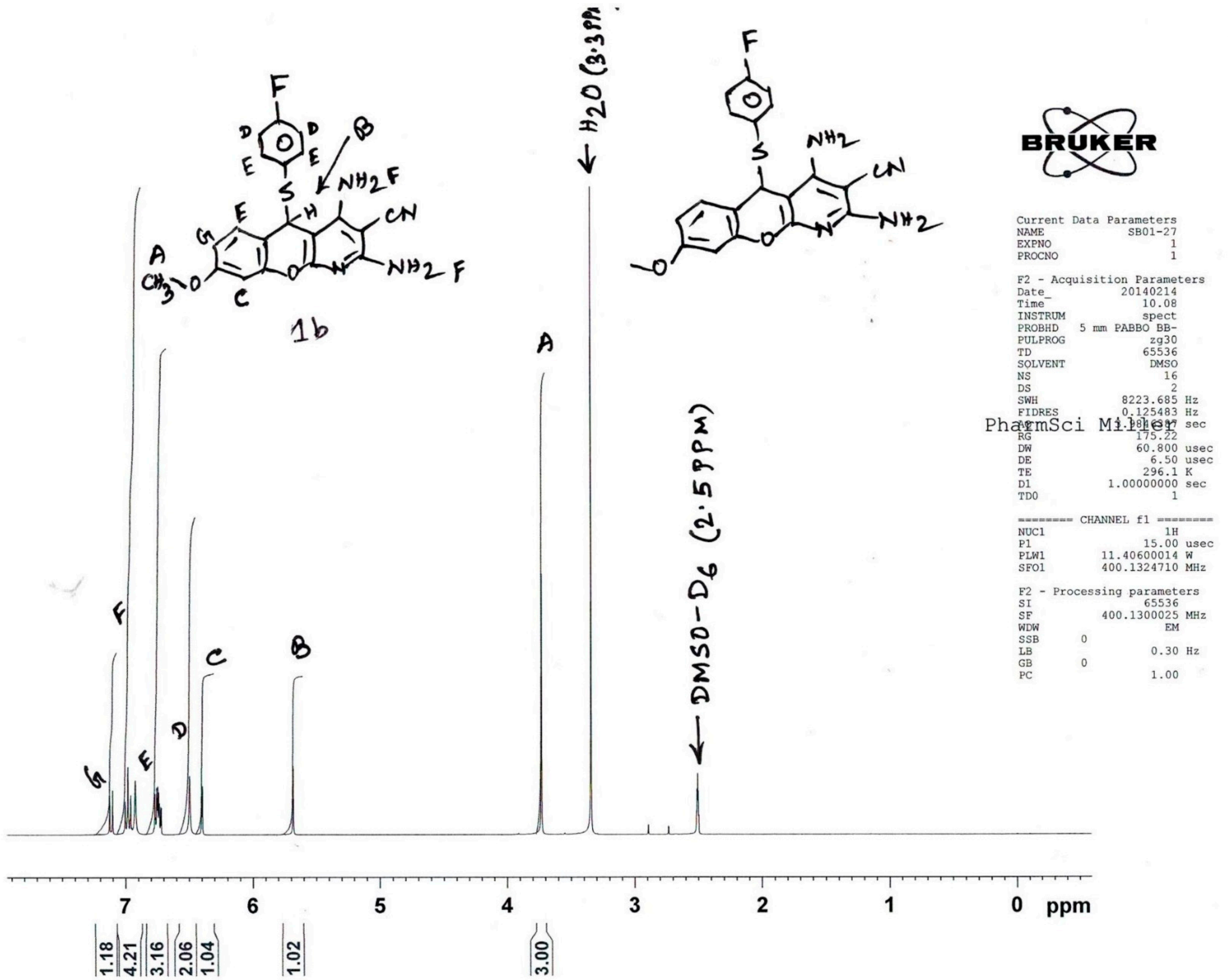

Figure S3. ^{1}H-NMR of **1b**.

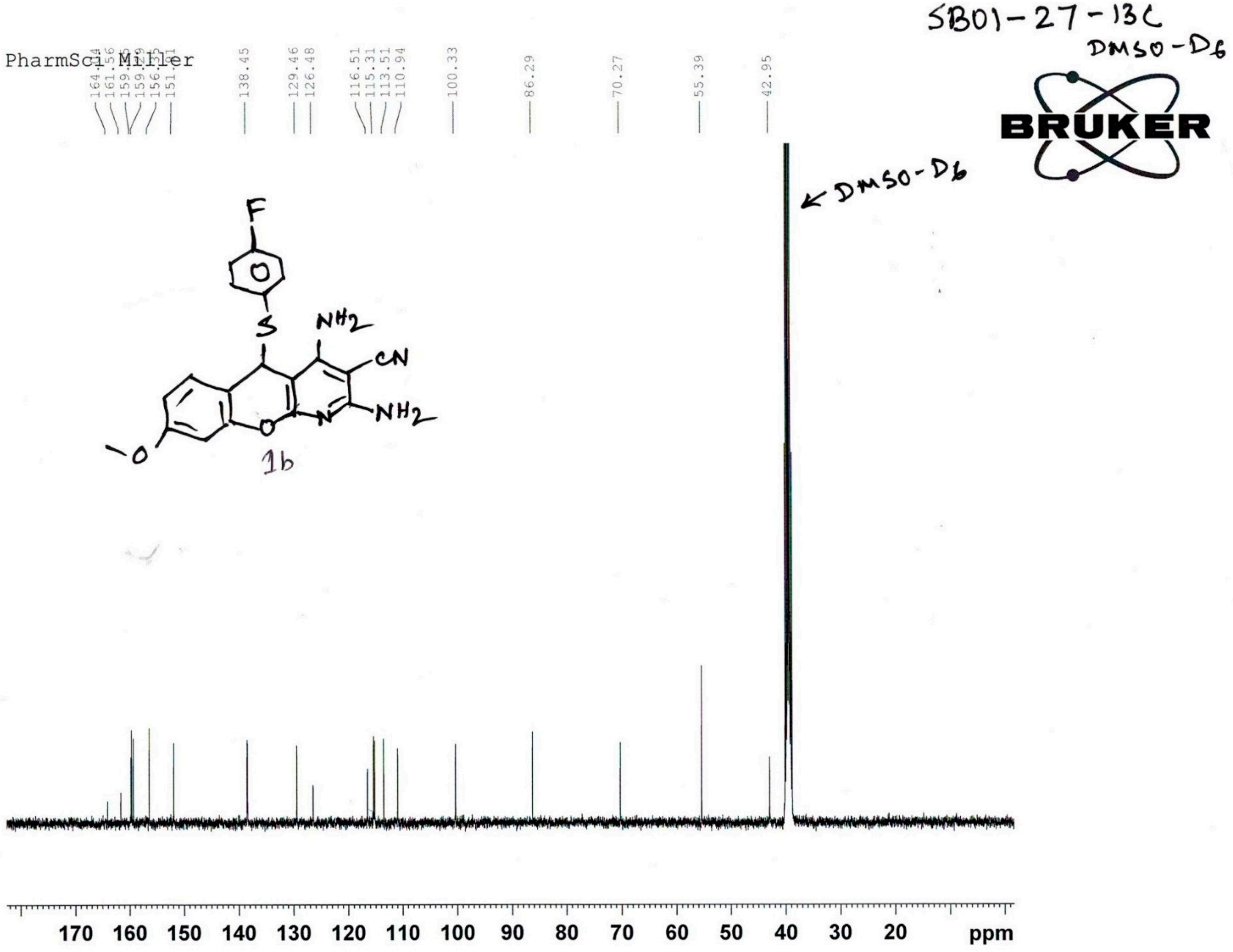

Figure S4. ^{13}C-NMR of **1b**.

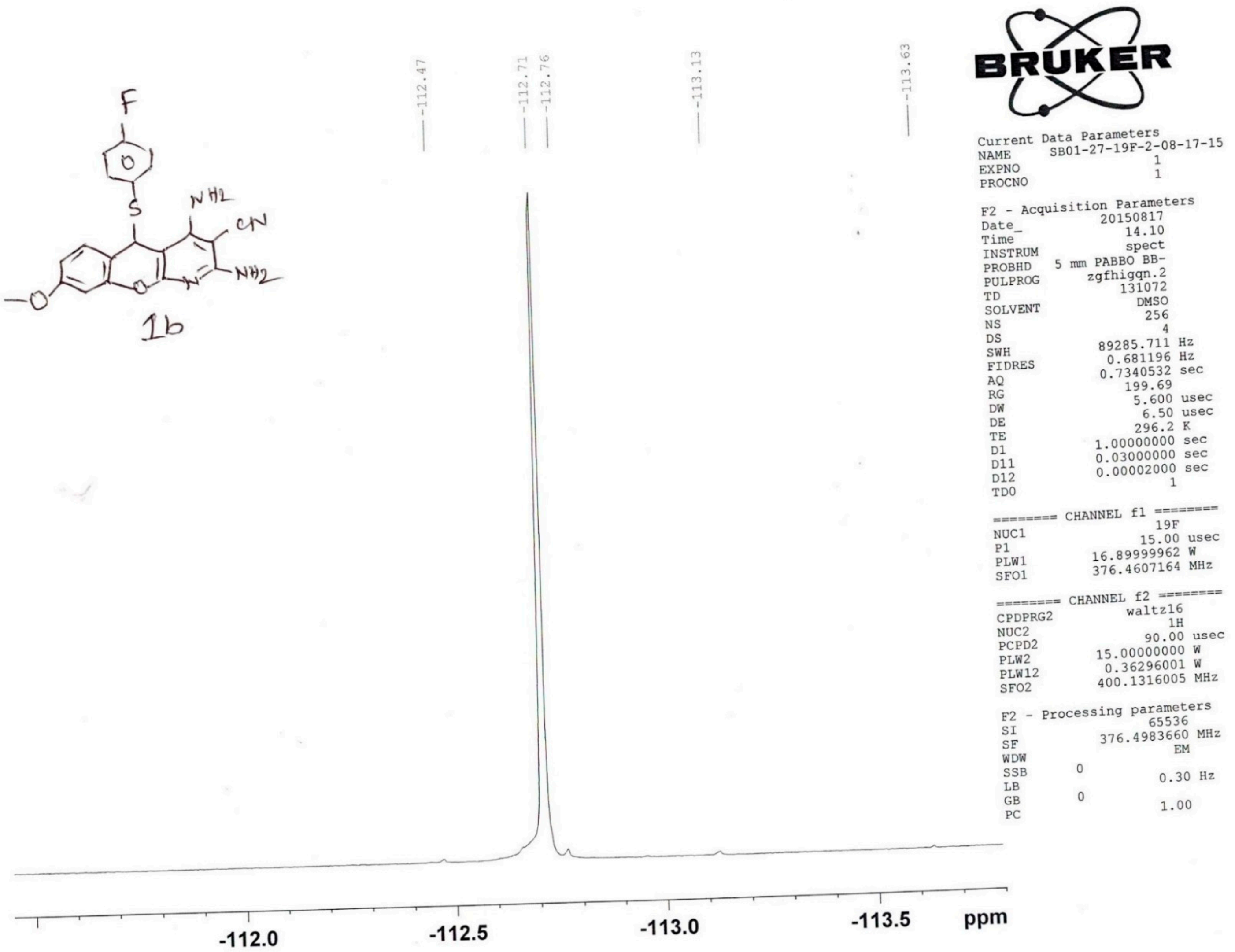

Figure S5. ^{19}F-NMR of **1b**.

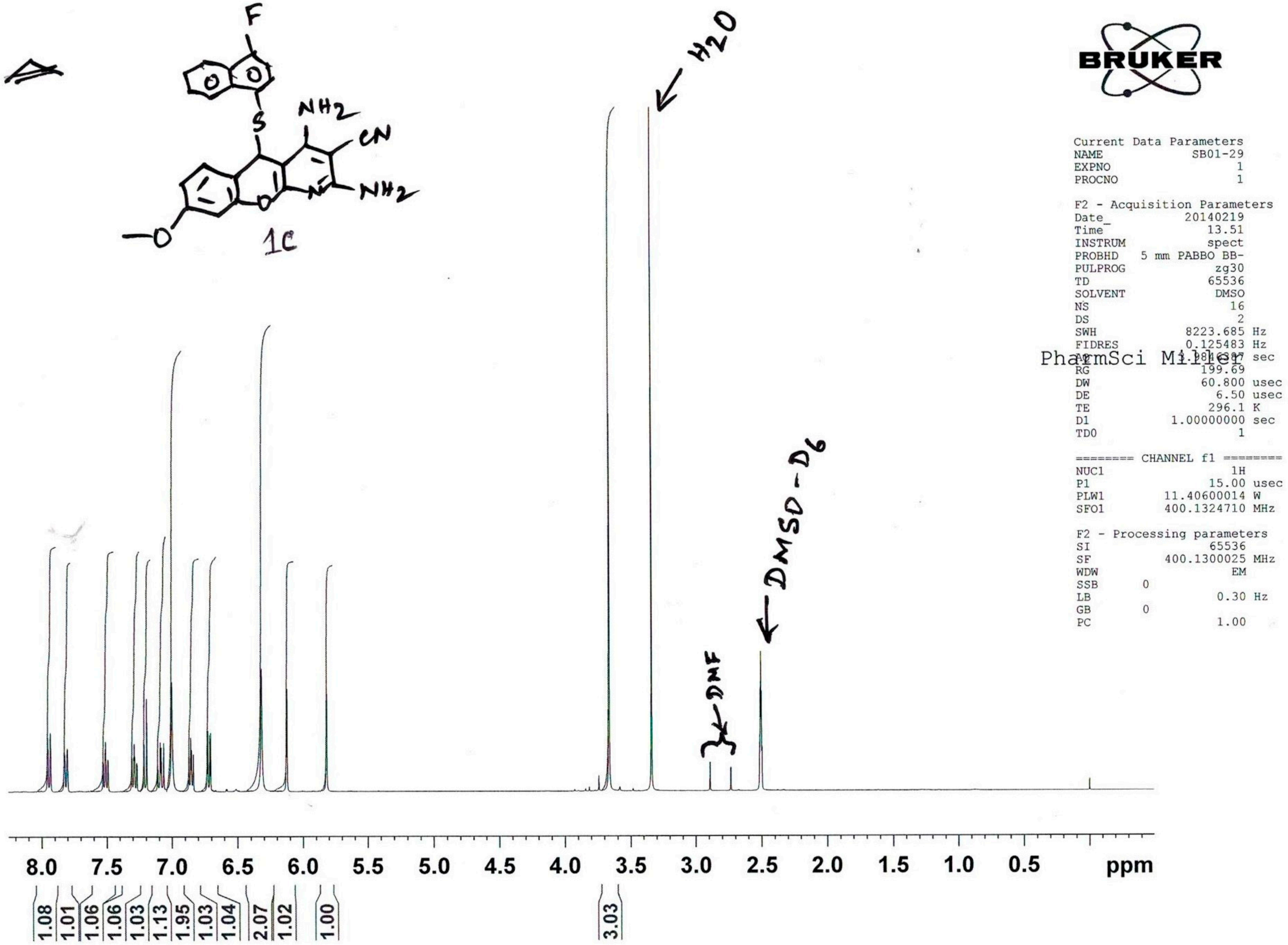

Figure S6. ^{1}H-NMR of **1c**.

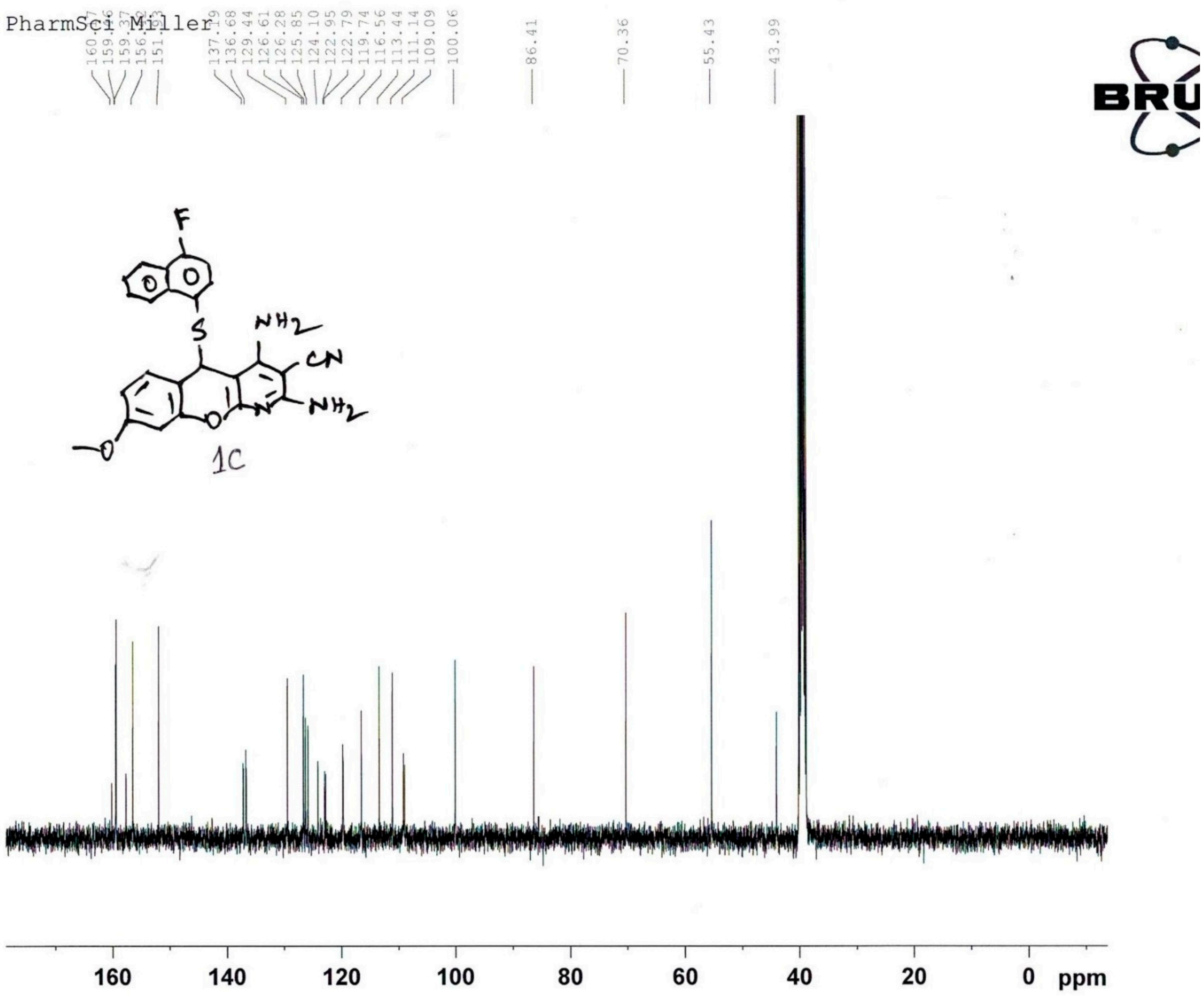

Figure S7. ^{13}C-NMR of **1c**.

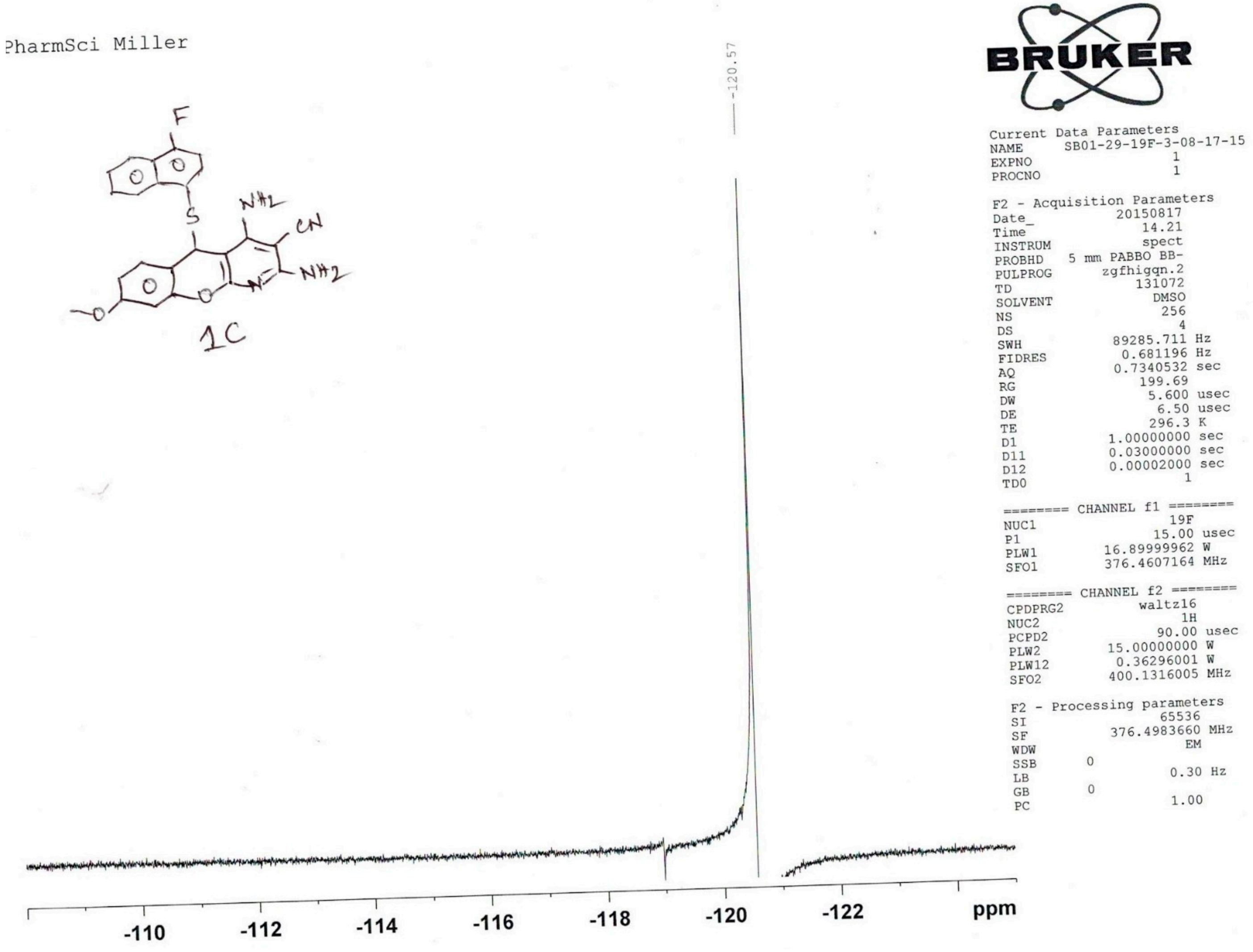

Figure S8. ^{19}F-NMR of **1c**.

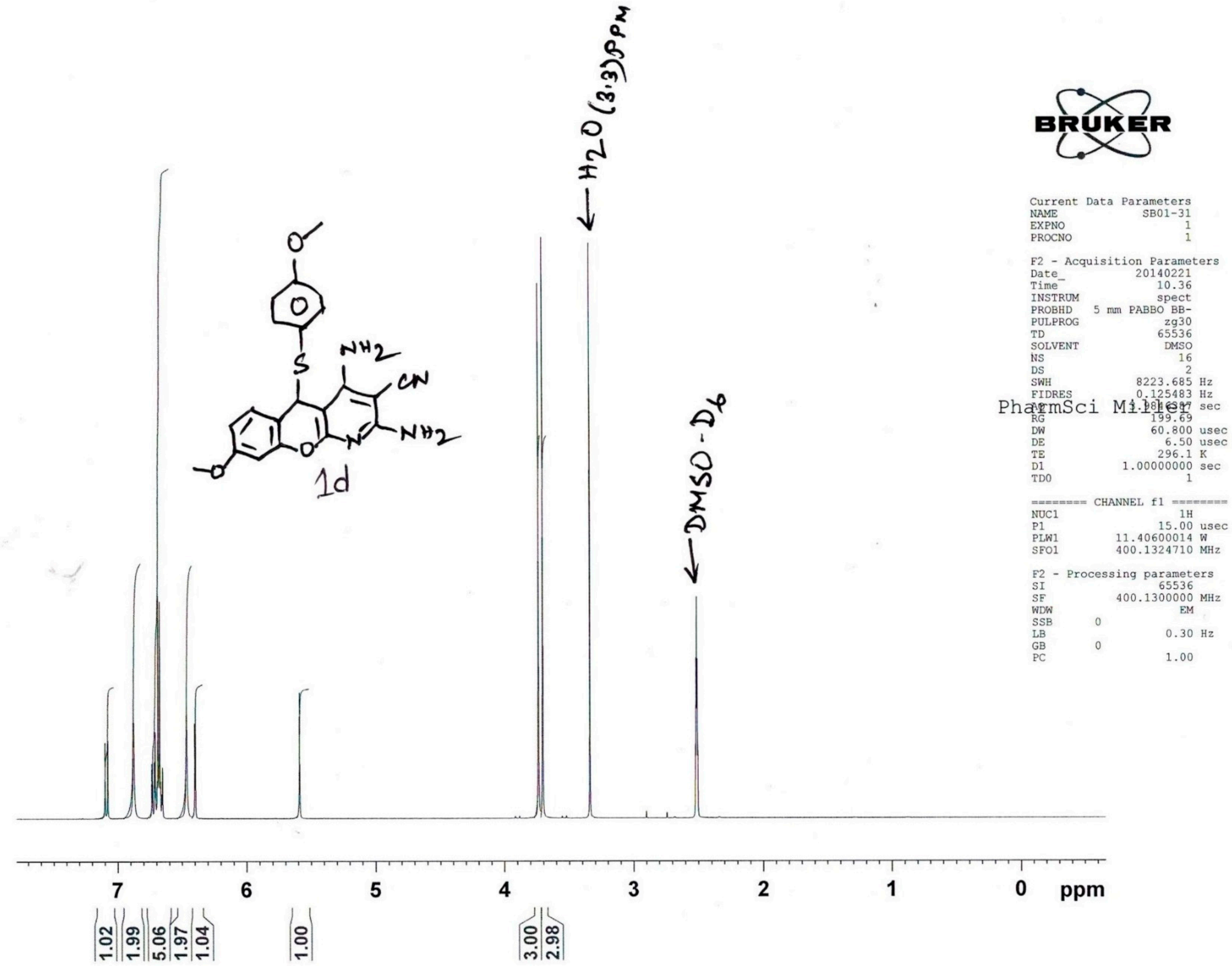

Figure S9. ^{1}H-NMR of **1d**.

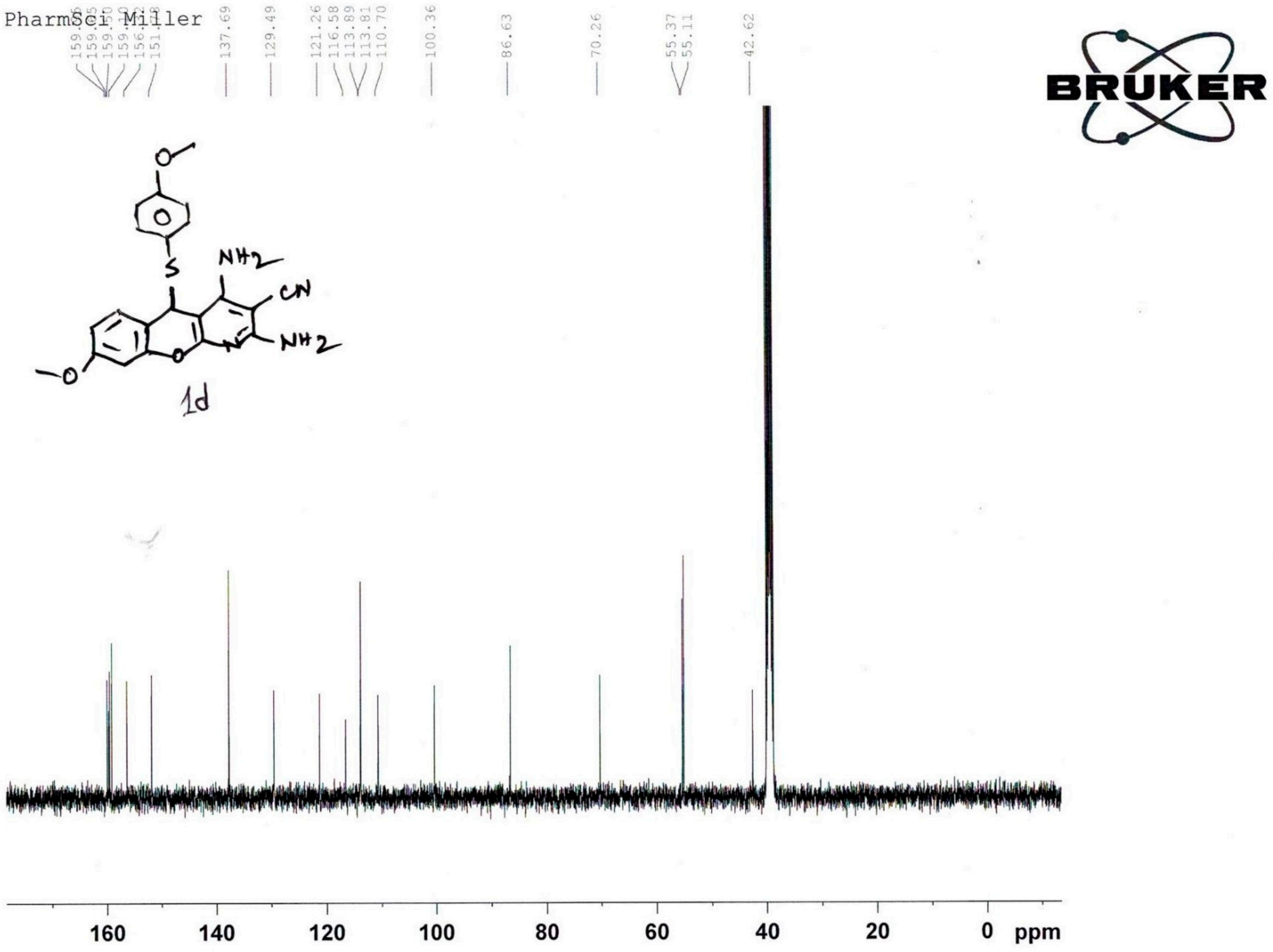

Figure S10. ^{13}C-NMR of **1d**.

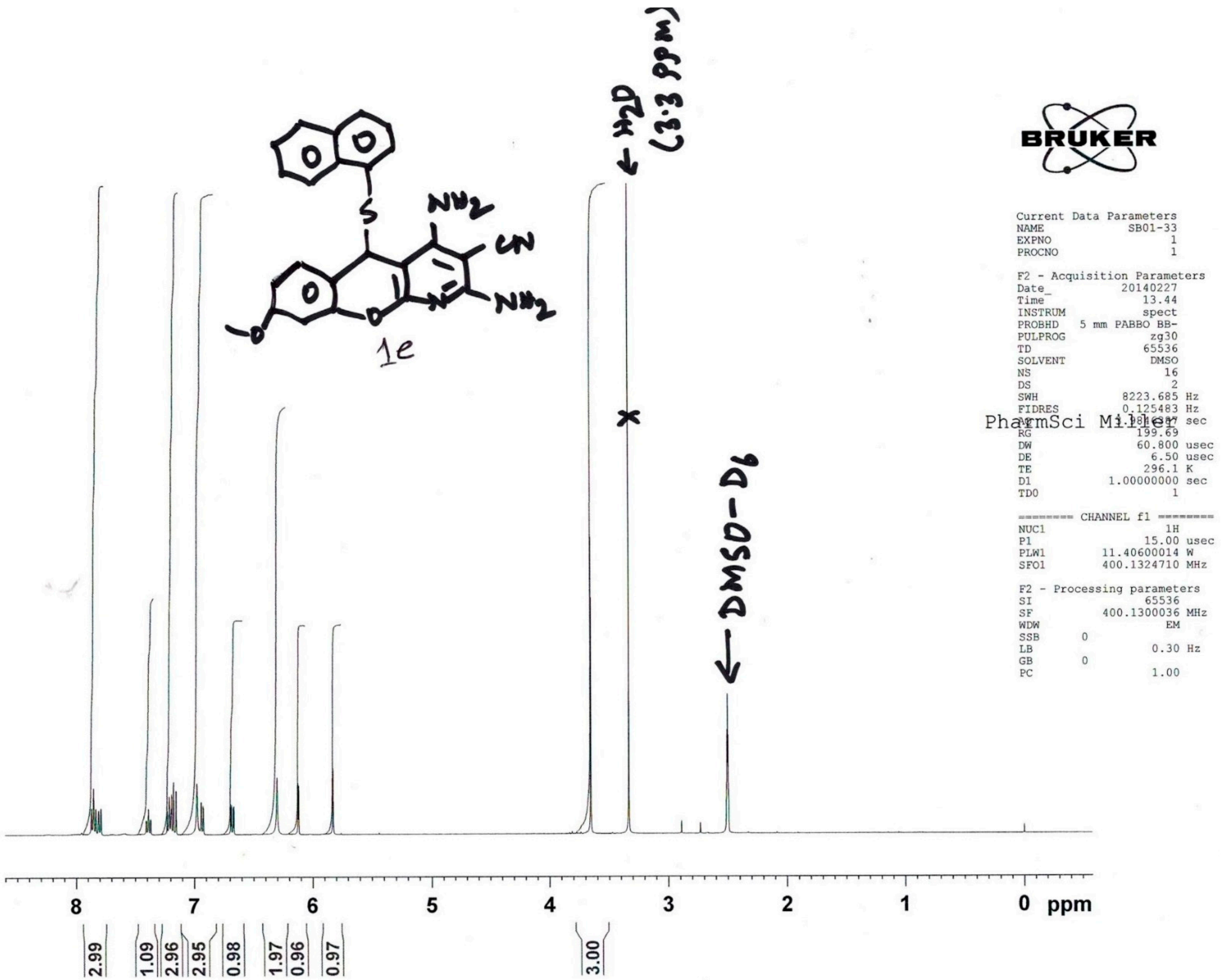

Figure S11. ^{1}H-NMR of **1e**.

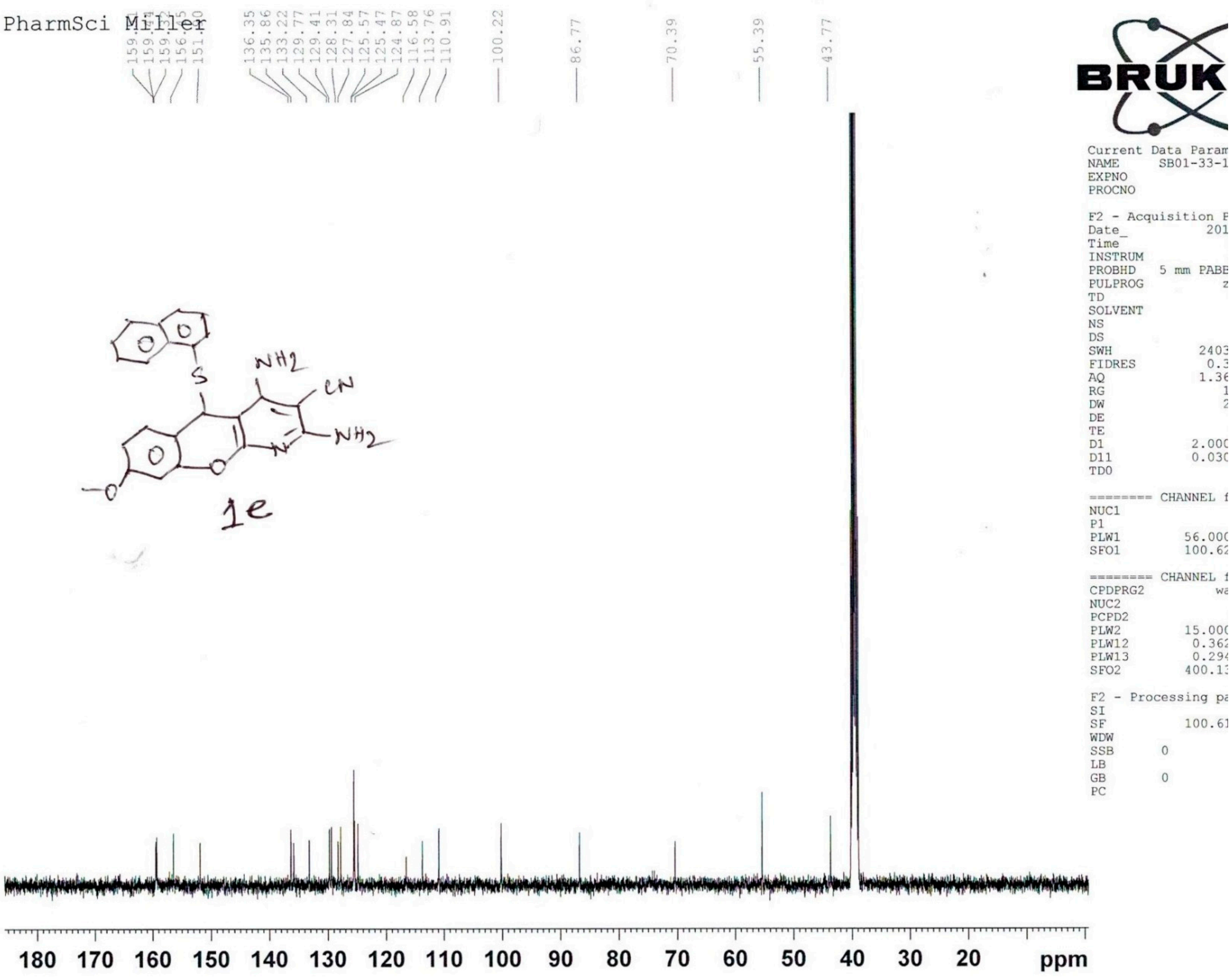

Figure S12. ^{13}C-NMR of **1e**.

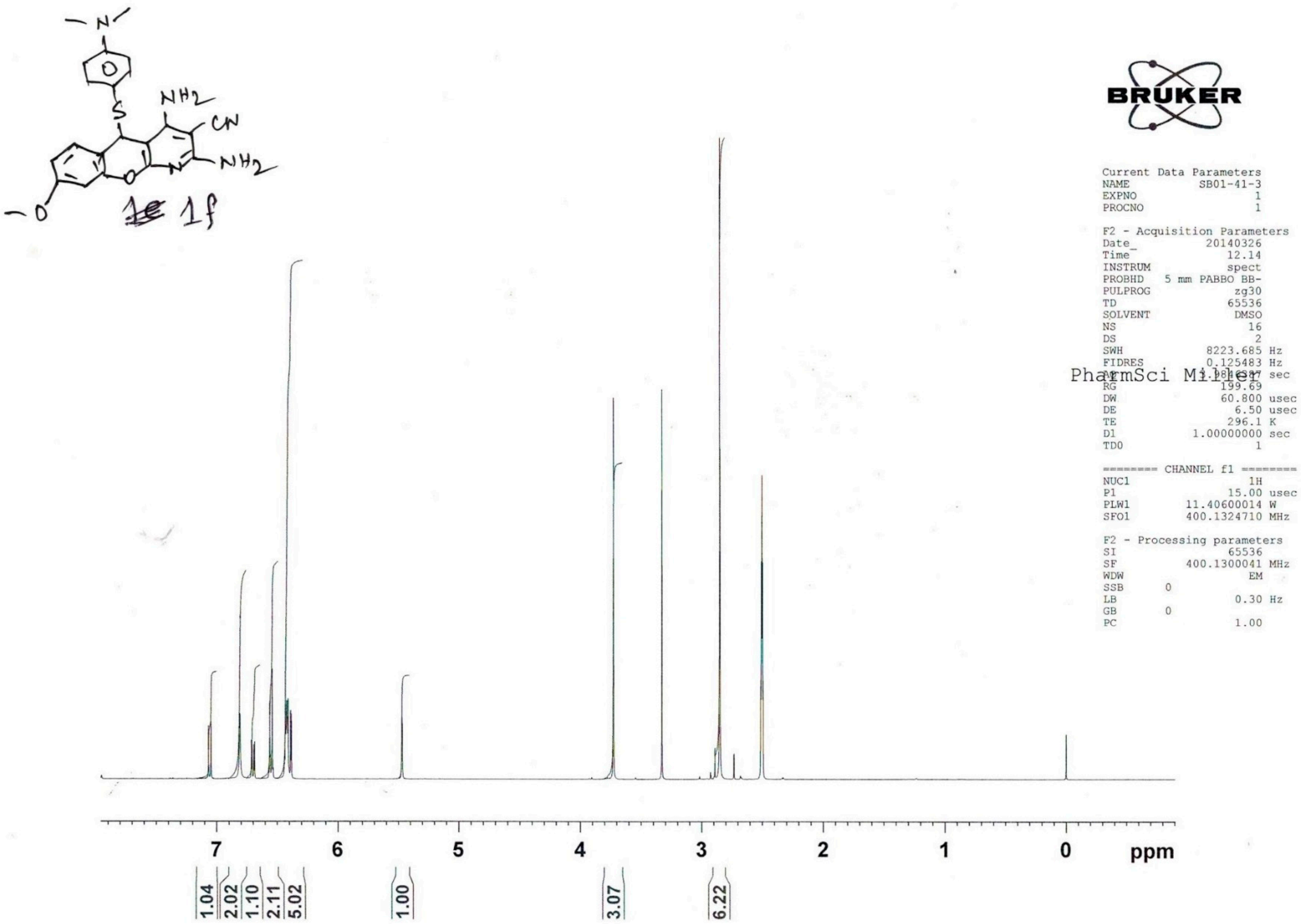

Figure S13. ^{1}H-NMR of **1f**.

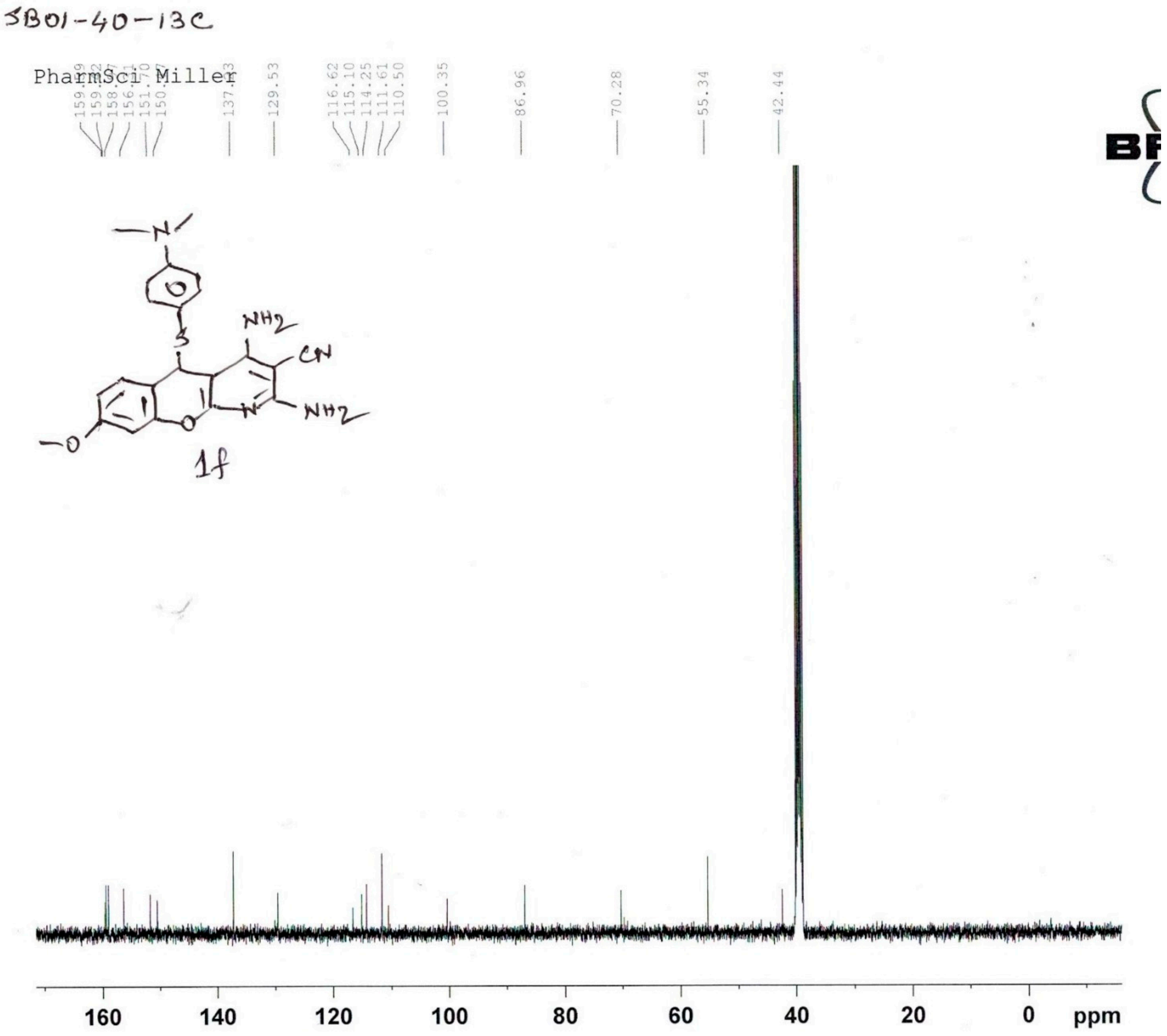

Figure S14. ^{13}C-NMR of **1f**.

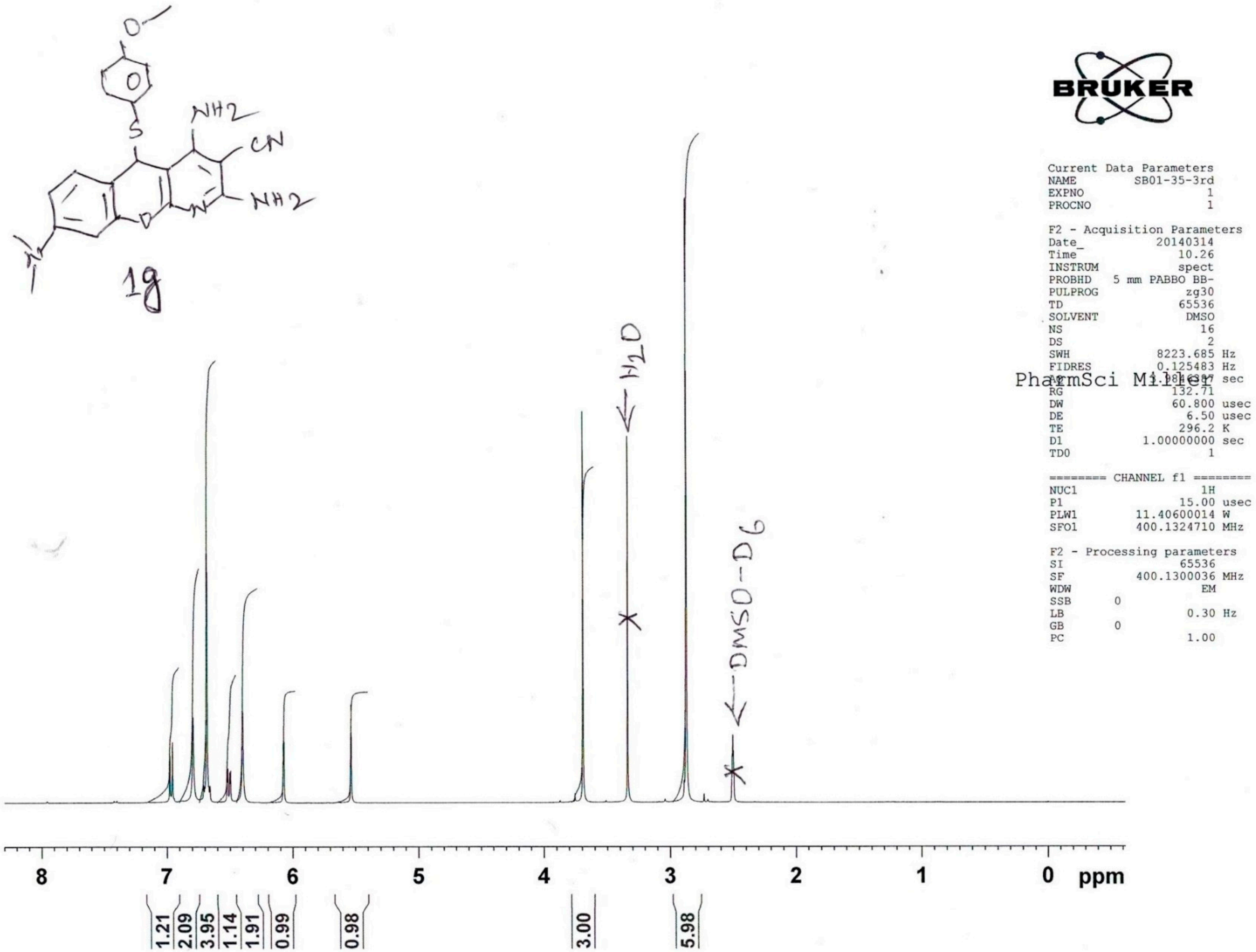

Figure S15. ^{1}H-NMR of **1g**.

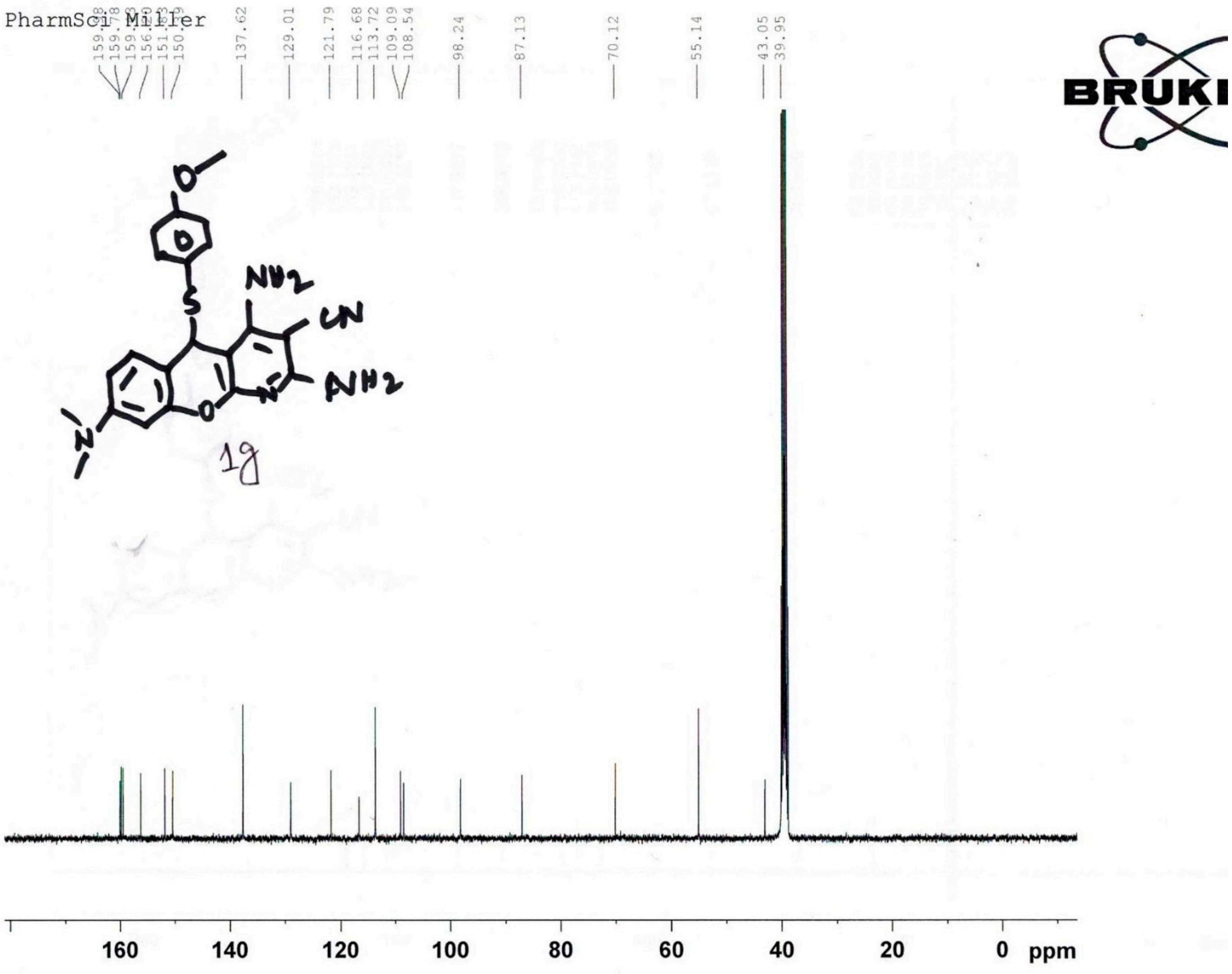

Figure S16. ^{13}C-NMR of **1g**.

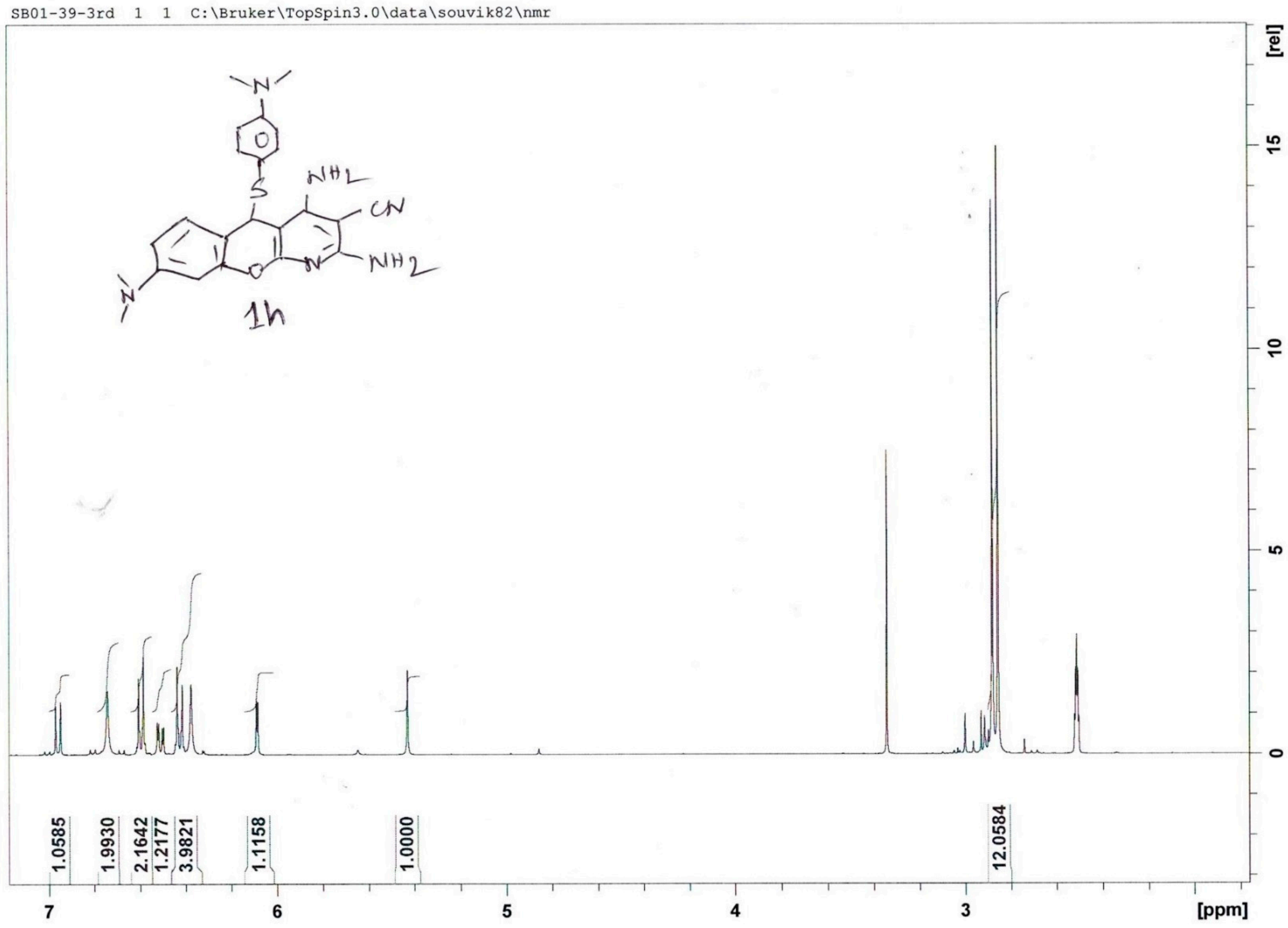

Figure S17. ^{1}H-NMR of **1h**.

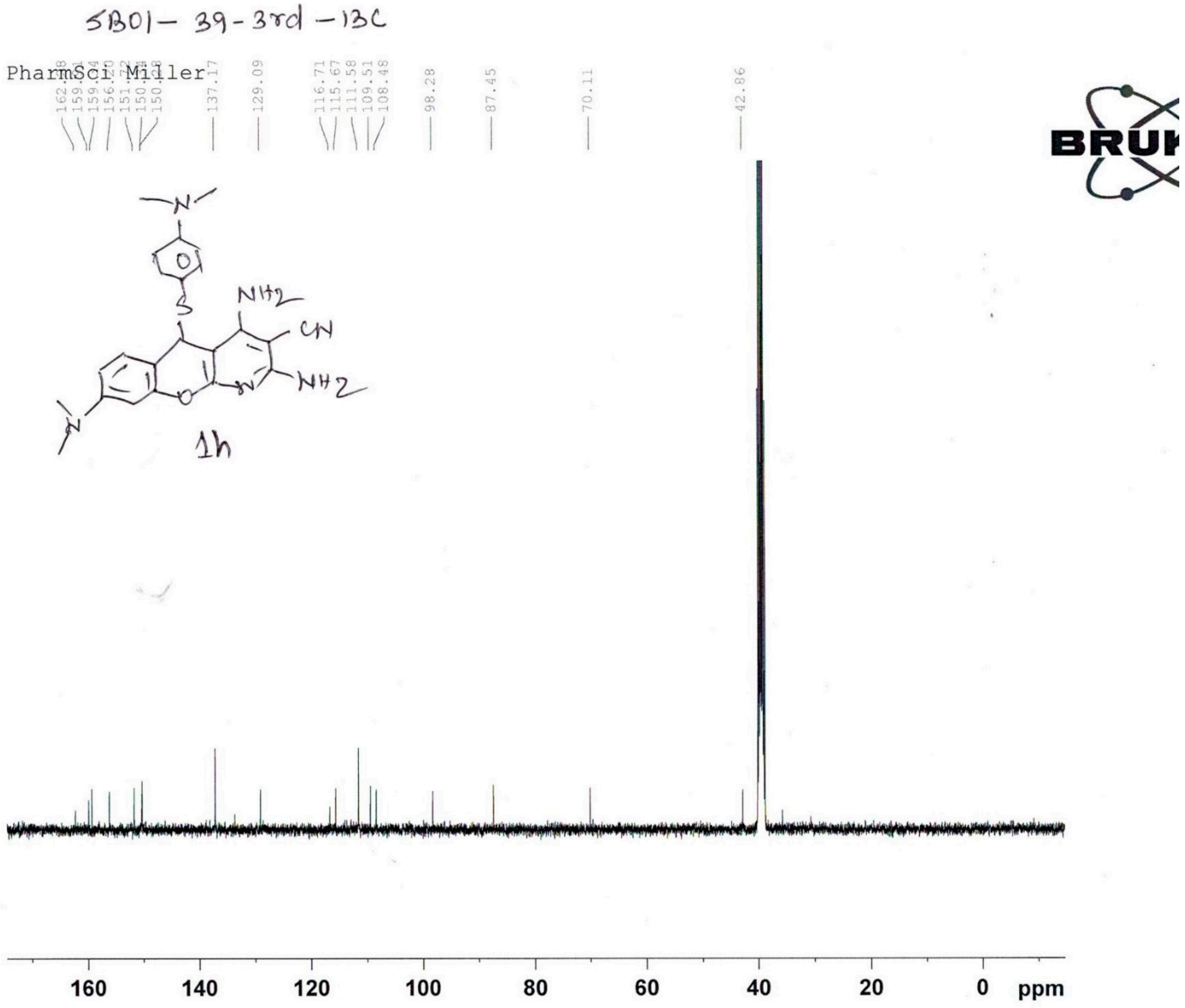

Figure S18. ^{13}C-NMR of **1h**.

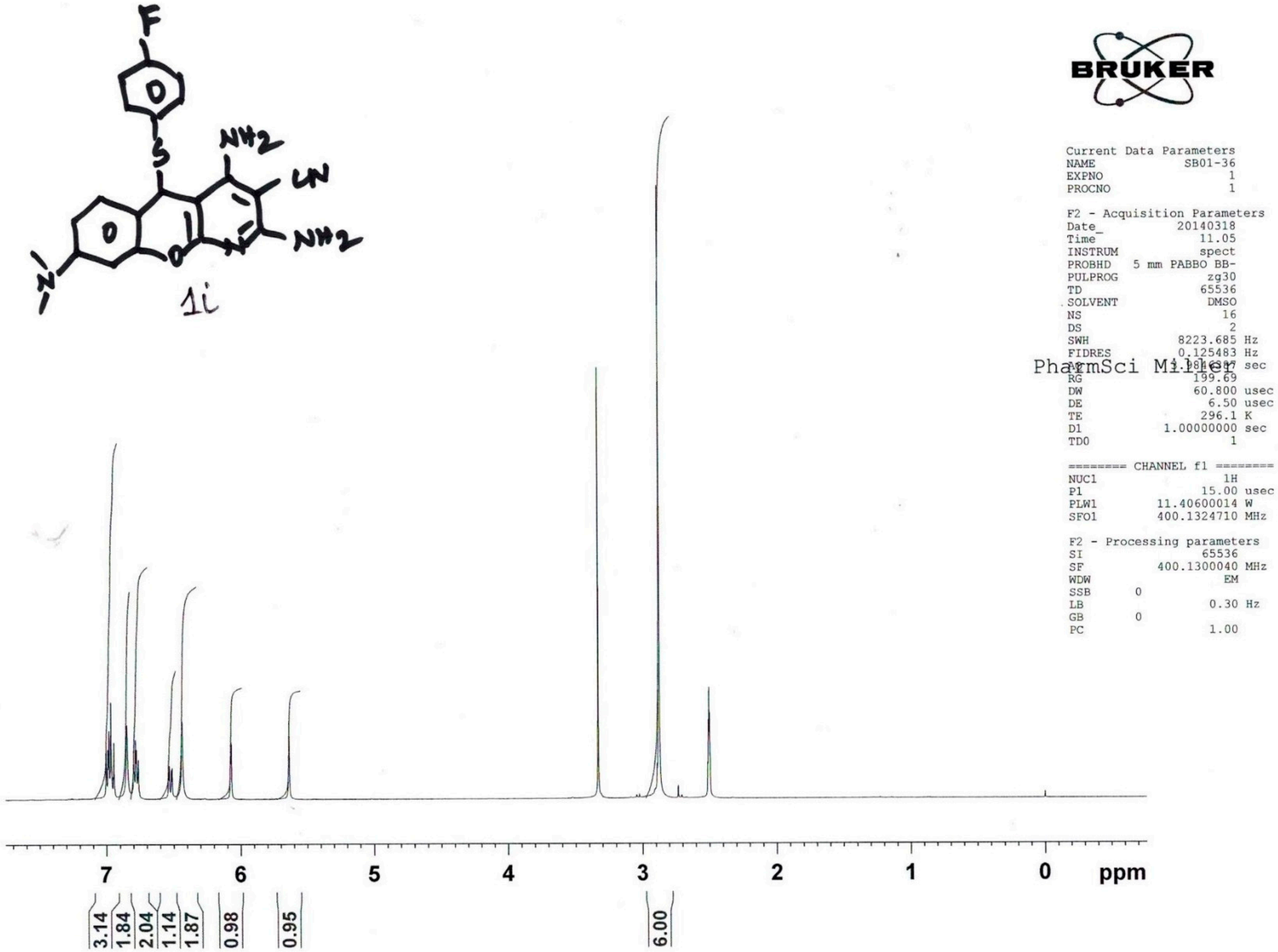

Figure S19. ^{1}H-NMR of **1i**.

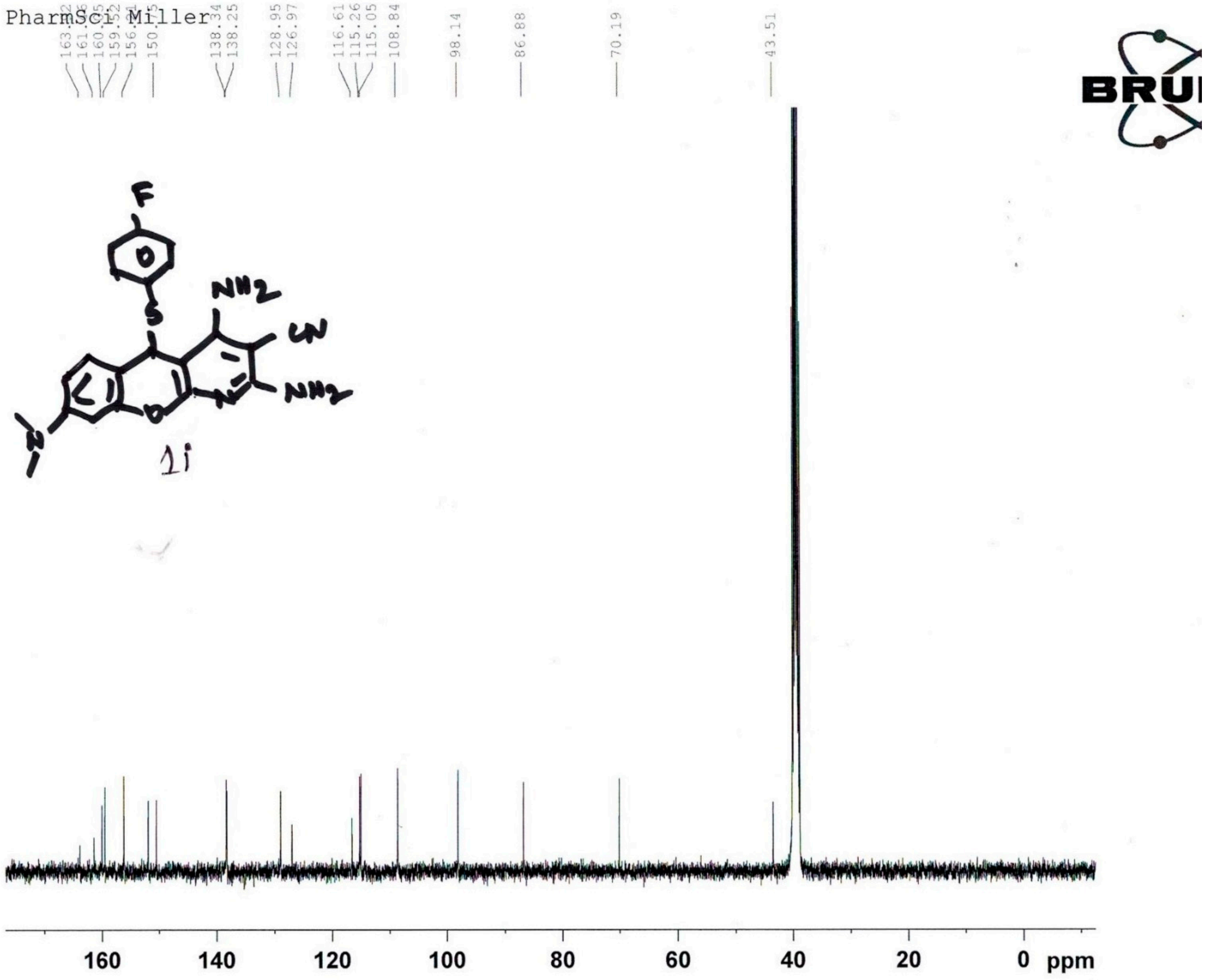

Figure S20. ^{13}C-NMR of **1i**.

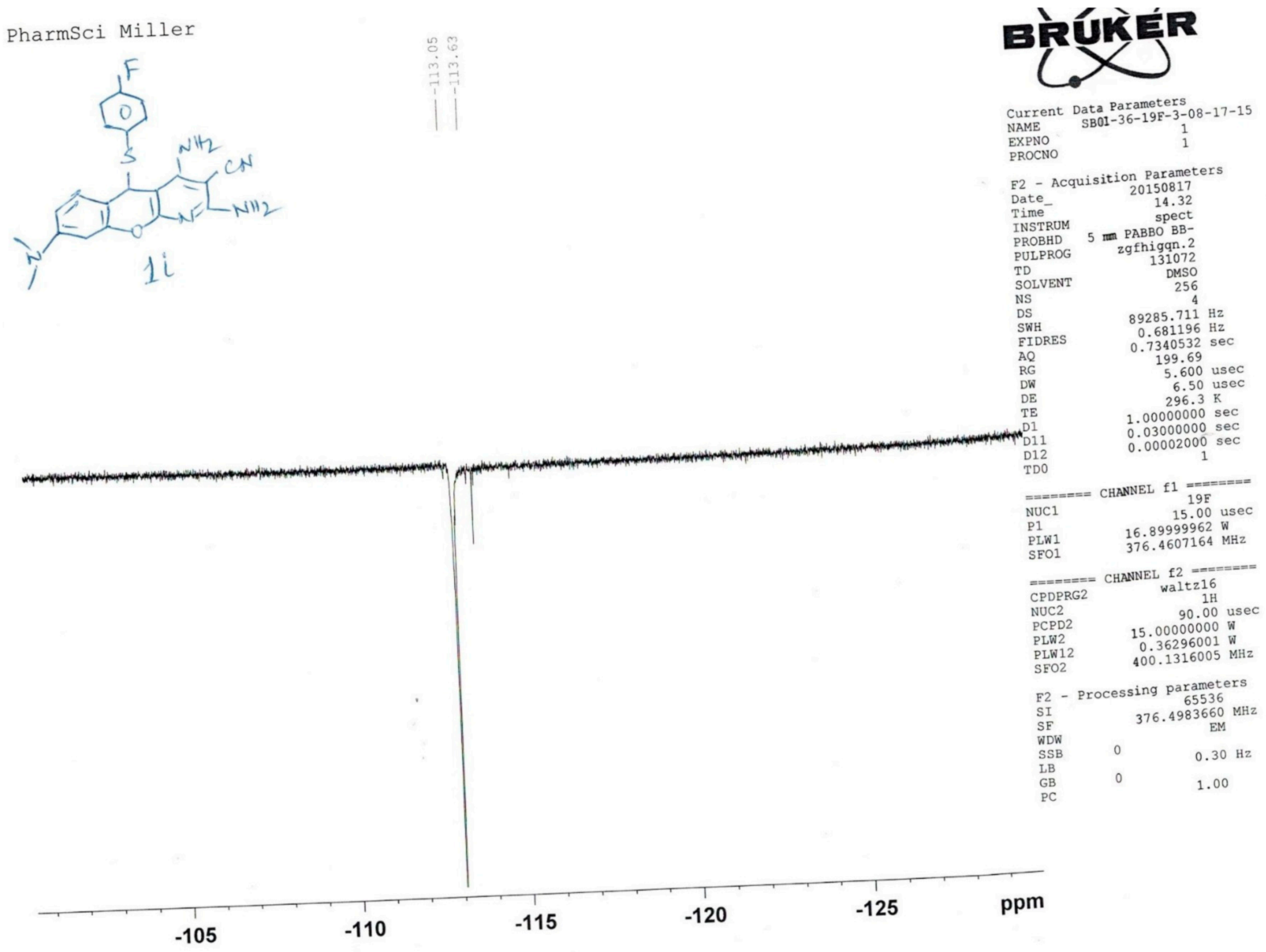

Figure S21. ^{19}F-NMR of **1i**.

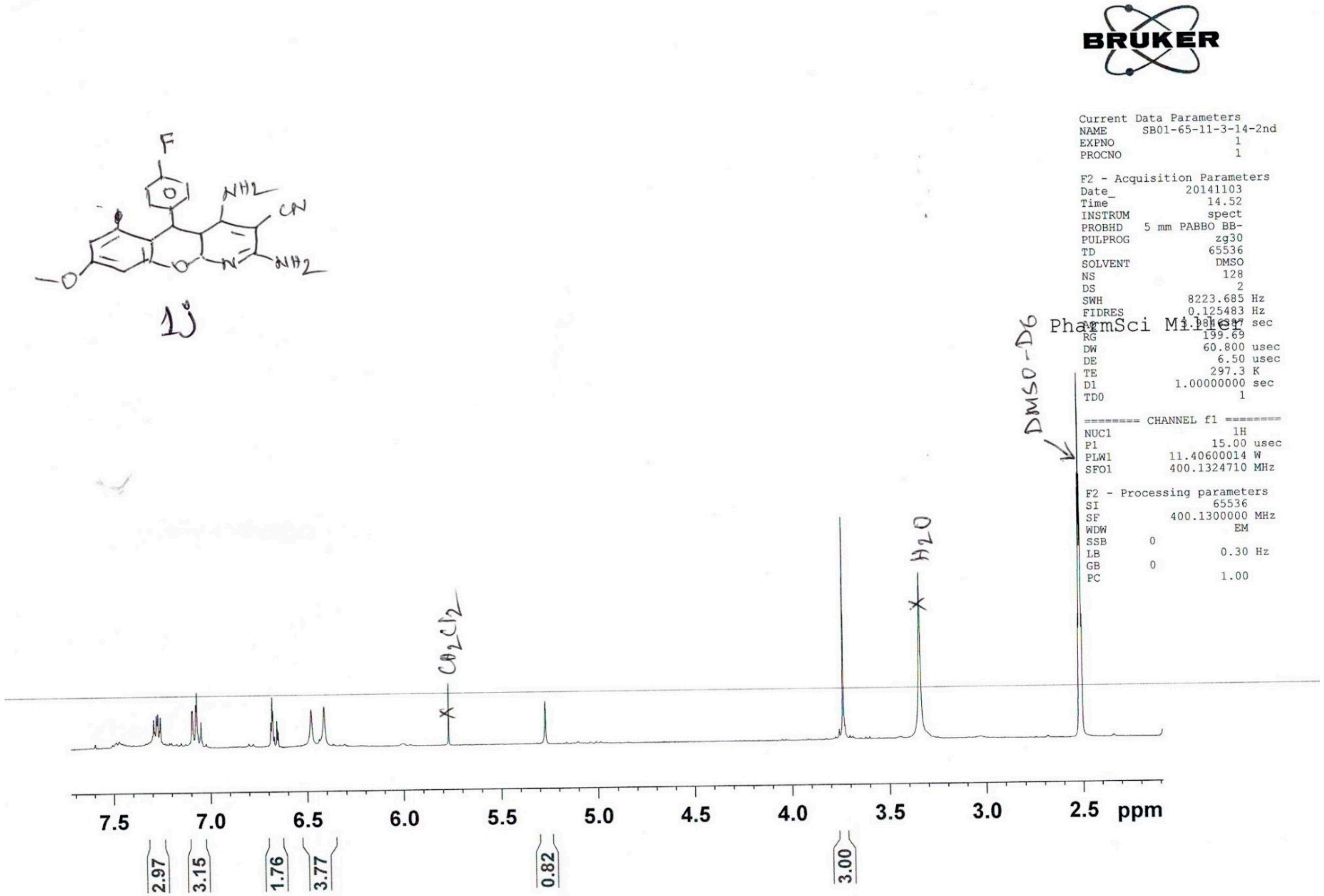

Figure S22. ^{1}H-NMR of **1j**.

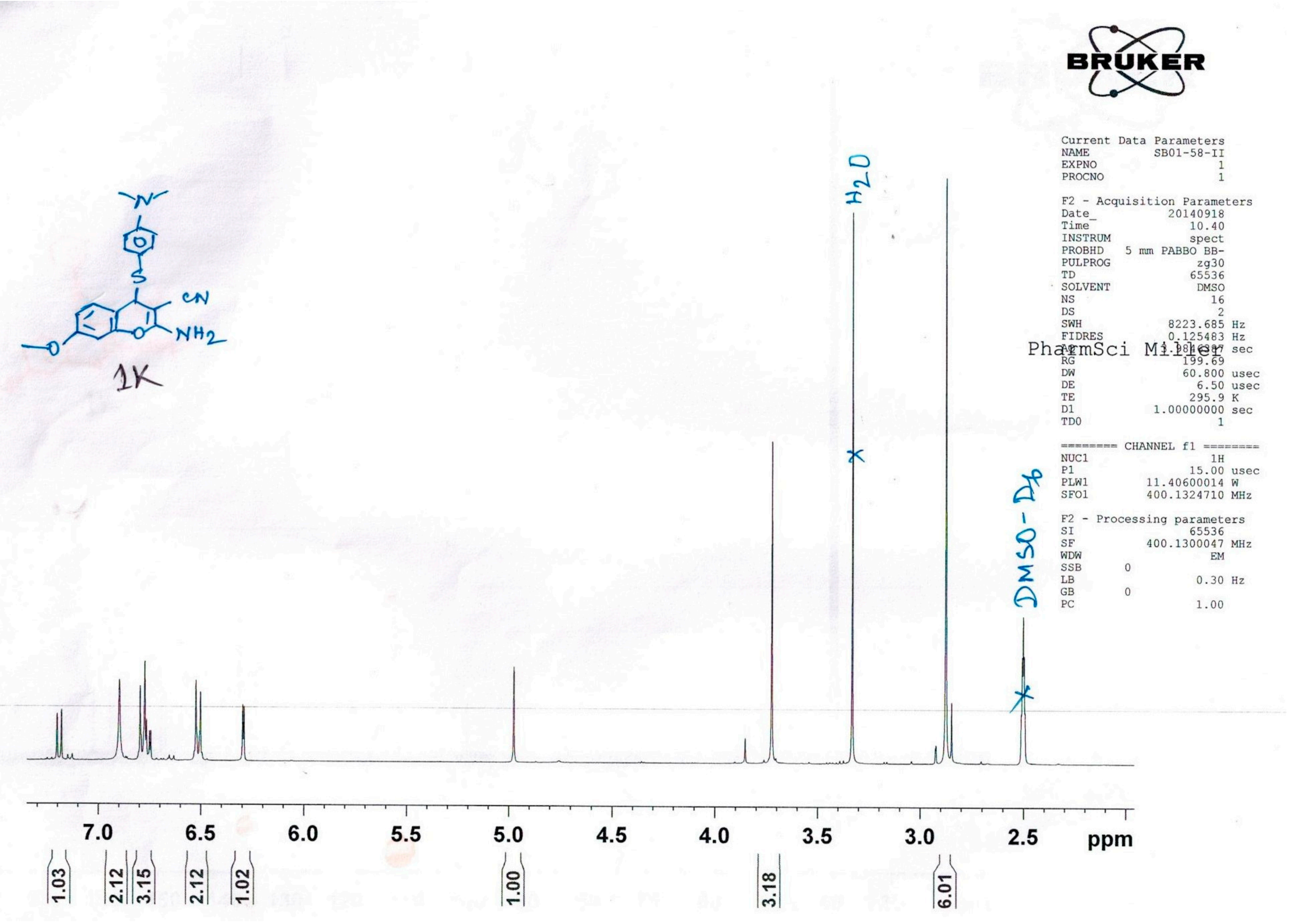

Figure S23. ^{1}H-NMR of **1k**.

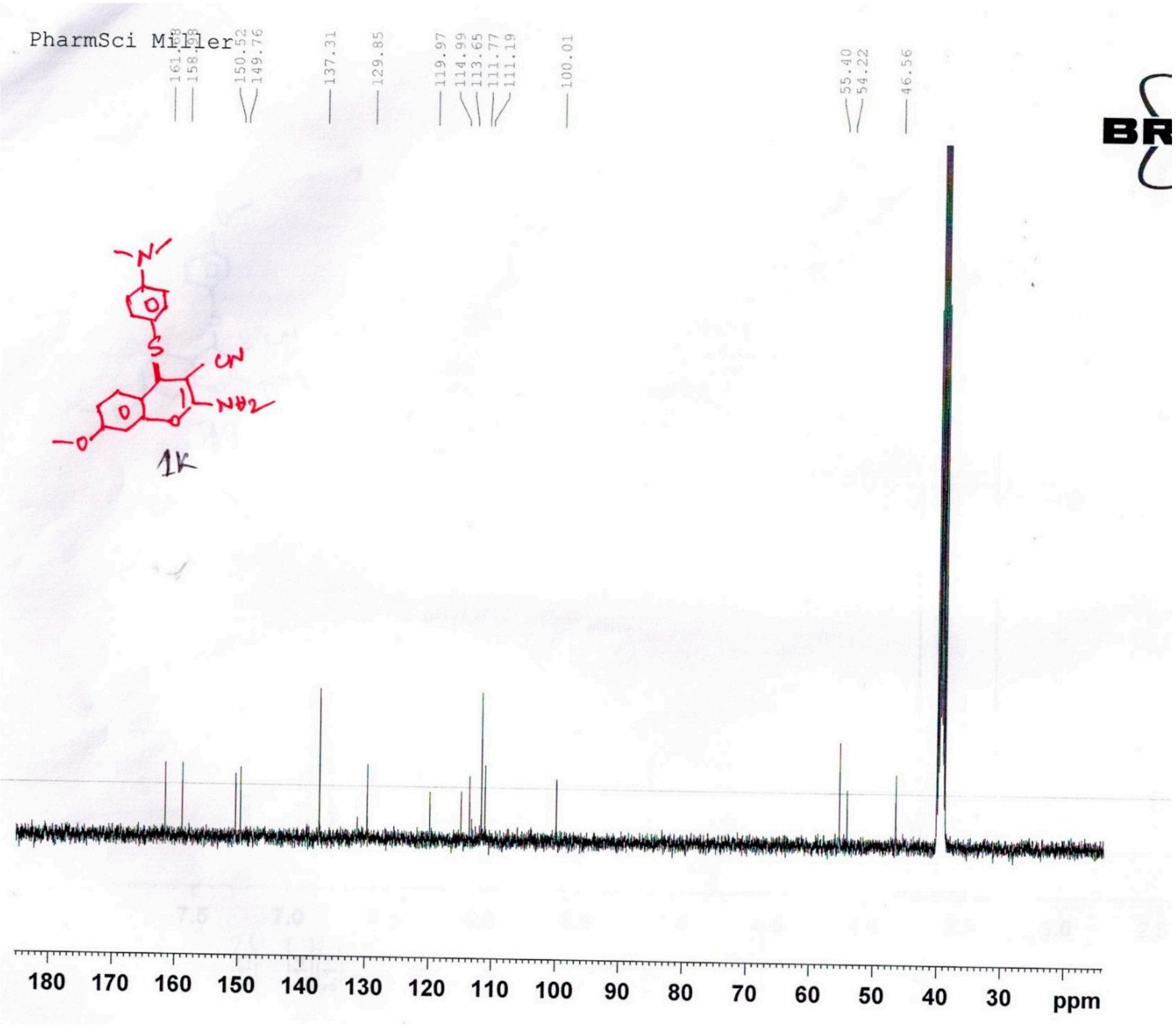

Figure S24. ^{13}C-NMR of **1k**.

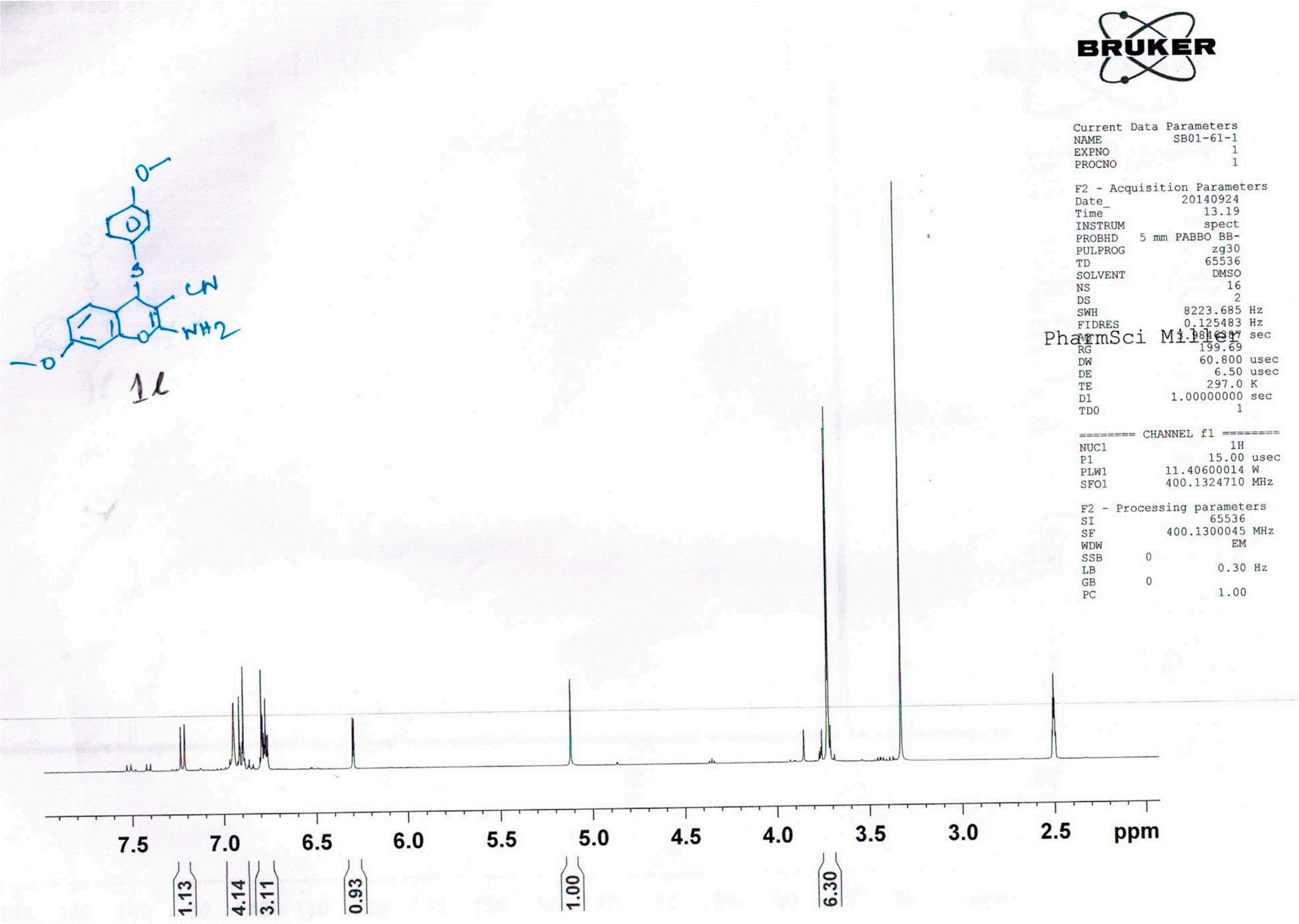

Figure S25. ^{1}H-NMR of **1l**.

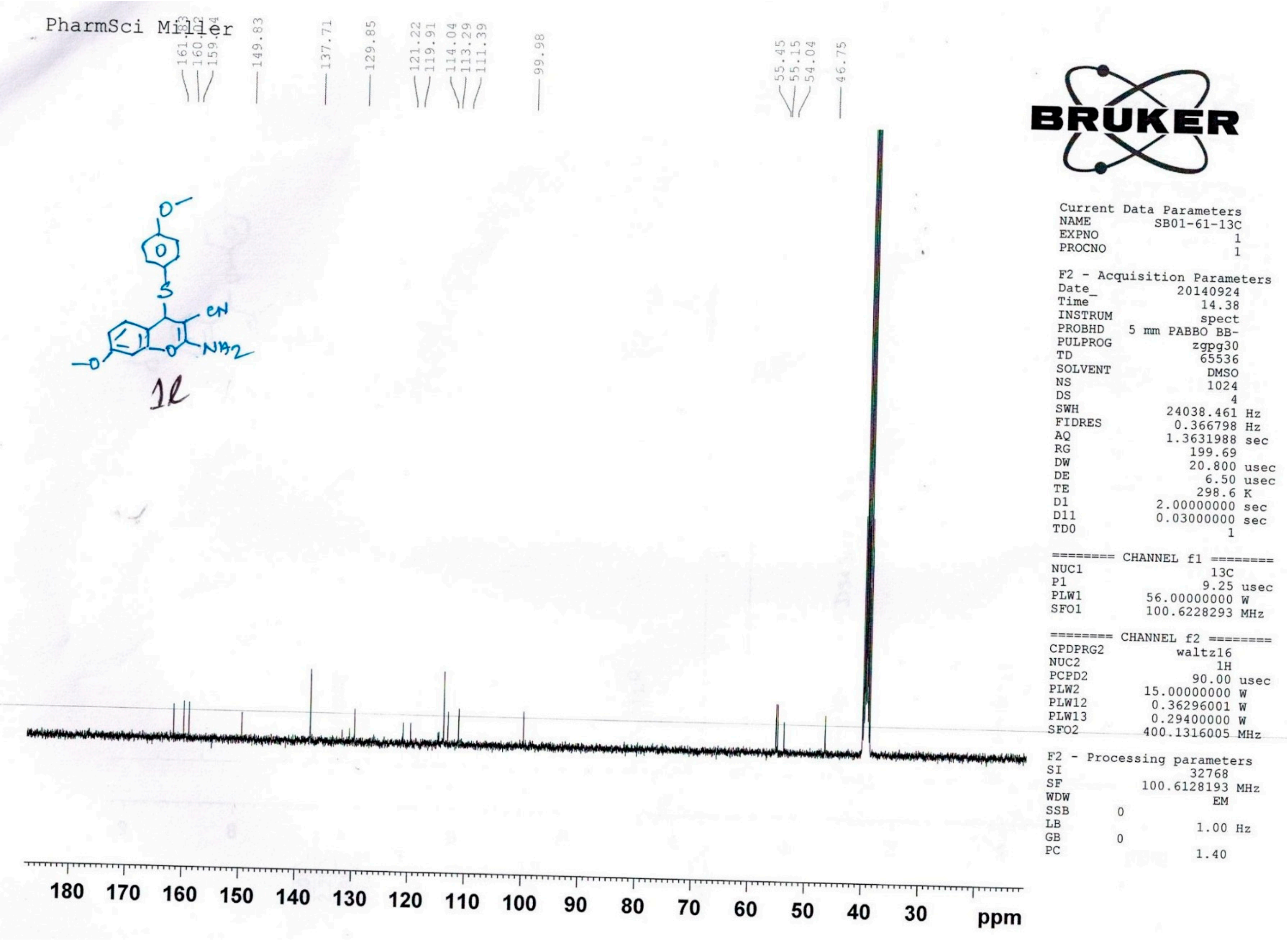

Figure S26. ^{13}C-NMR of **1l**.

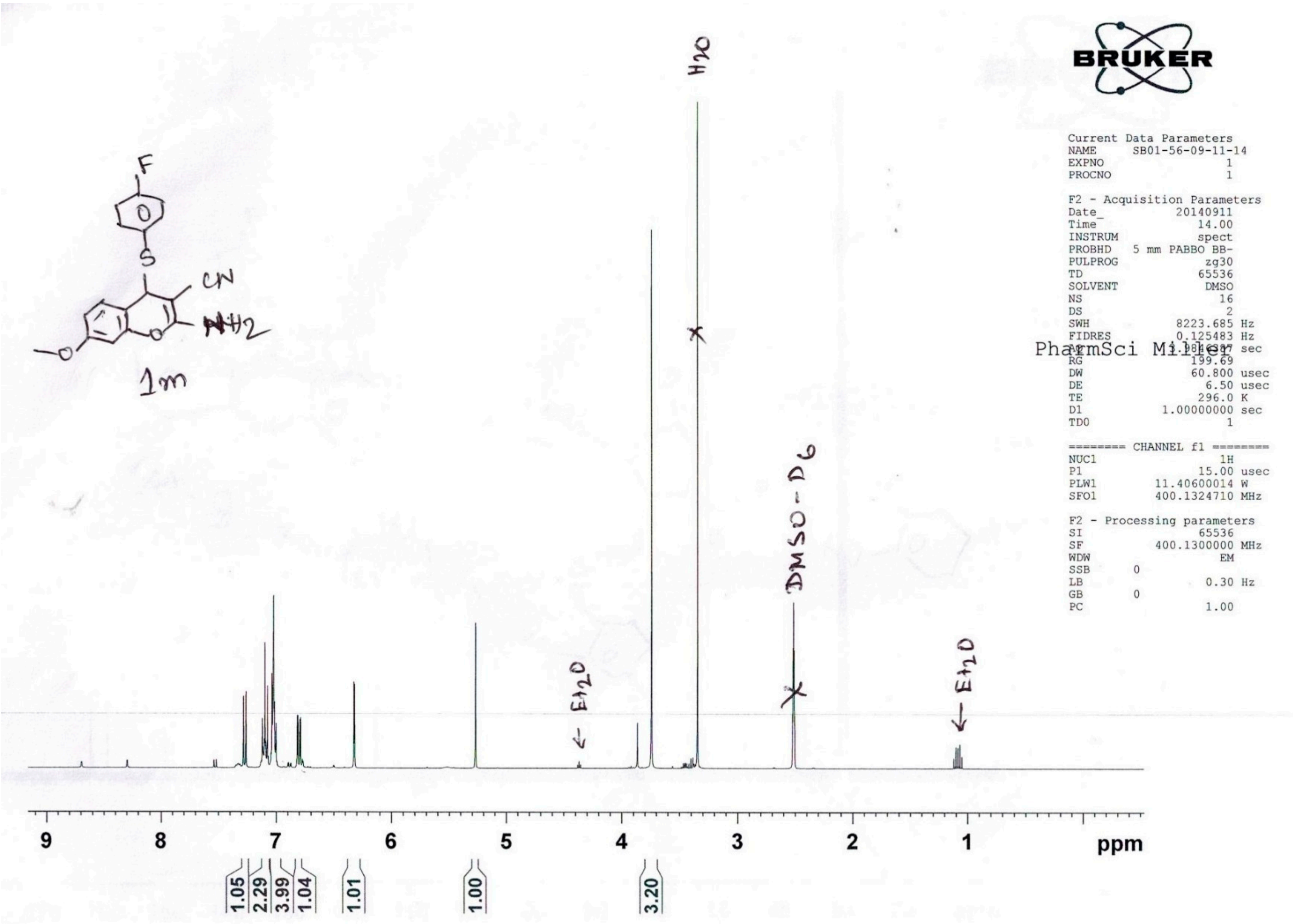

Figure S27. ^{1}H-NMR of **1m**.

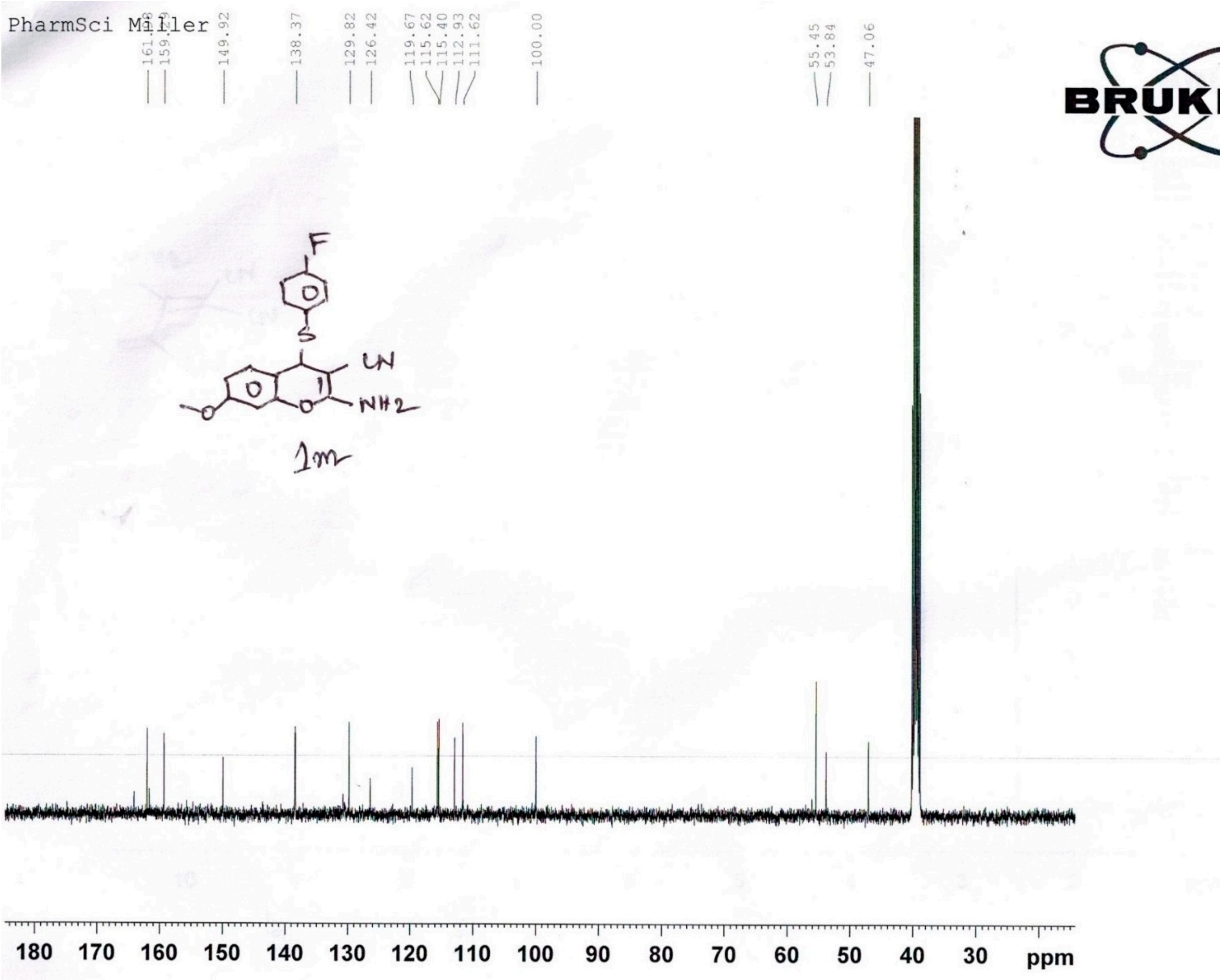

Figure S28. ^{13}C-NMR of **1m**.

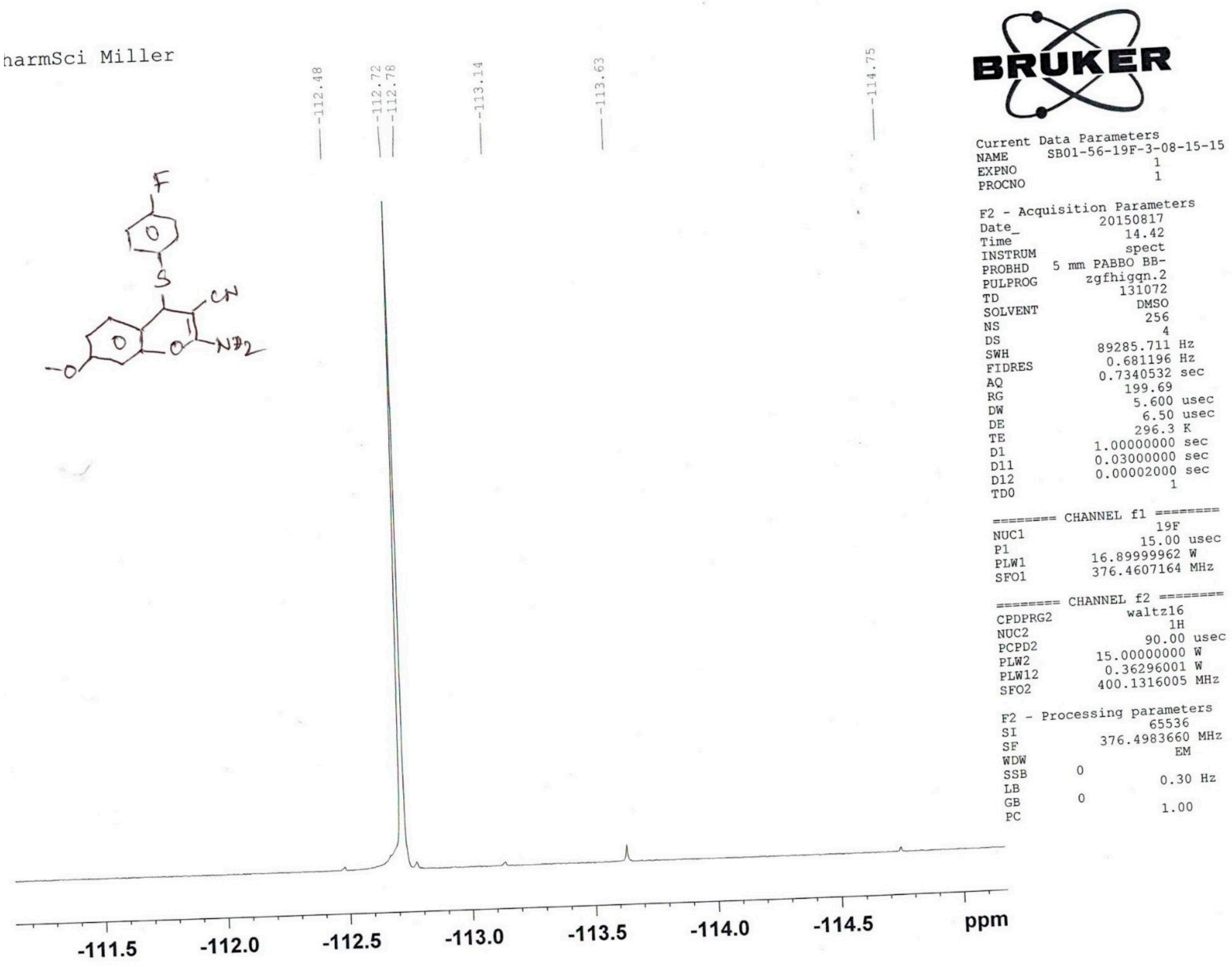

Figure S29. ^{19}F-NMR of **1m**.
